# Insights into phylogenetic relationships of *Veronica* species (Plantaginaceae) based on comparative chloroplast genomics

**DOI:** 10.3389/fpls.2026.1885890

**Published:** 2026-08-18

**Authors:** Ying Huang, Shihao Jiang, Yanru Zhang, Xingyu Du, Qinyi Wang, Jiazhen Wu, Xia Pan, Zhixi Fu, Jing Zhou

**Affiliations:** 1 College of Life Sciences, Sichuan Normal University, Chengdu, China; 2 Key Laboratory of Land Resources Evaluation and Monitoring in Southwest, Sichuan Normal University, Ministry of Education, Chengdu, China; 3 Sichuan Guoce Testing Technology Co., Ltd., Chengdu, China

**Keywords:** chloroplast genome, divergence time, phylogenetic relationship, Plantaginaceae, *Veronica*

## Abstract

**Introduction:**

Veronica L. is one of the most species-rich genera in Plantaginaceae and several species have medicinal, horticultural, or ecological value.

**Methods:**

In this study, the complete chloroplast genomes of three Veronica species were assembled and annotated using Illumina sequencing data.

**Results:**

The plastomes exhibited a typical quadripartite structures, with total lengths of 150,202 bp for Veronica biloba L., 151,159 bp for Veronica ciliata Fisch. and 151,098 bp for Veronica vandellioides Maxim. Each genome contained 130–132 unique genes, including 86–87 protein-coding genes, 36–37 tRNA genes, and 8 rRNA genes. Comparative analyses of 24 Veronica plastomes indicated that the IR/SC junctions were largely conserved, although slight boundary shifts occurred around rps19, ndhF, and ycf1. Forward, palindromic, complement, and reverse repeats were detected, and A/T mononucleotide repeats were the dominant SSR type. Nucleotide diversity analysis identified rpl32-trnL, trnK-rps16, rpl32, ycf1, ndhF, accD, matK, and rpoB as highly variable regions. Phylogenetic analyses recovered Veronica as a well-supported monophyletic lineage and clarified the plastid positions of the three newly sequenced species. Divergence time estimation suggested that the estimation suggested of Veronica was around 14.9 Ma, with V. biloba, V. ciliata and V. vandellioides diverging approximately 3.9 Ma, 0.6 Ma, and 6.9 Ma, respectively.

**Discussion:**

Because the analyses were based on plastid genomes, the inferred topology should be interpreted as chloroplast phylogenetic evidence rather than a complete species-history reconstruction. These results provide plastome resources and molecular evidence for taxonomy, species identification, and future evolutionary studies of Veronica.

## 1 Introduction

Plantaginaceae belongs to Lamiales and serves as a key group for systematic classification and evolutionary studies of angiosperms (Olmstead et al., 2001; Albach et al., 2005b; Refulio-Rodriguez and Olmstead, 2014; Chase et al., 2016). *Veronica* L. is the most species-rich genus in Plantaginaceae in its current circumscription, with approximately 450 described species (Albach et al., 2004a; Fischer, 2004). It is widely distributed in temperate, subtropical, and frigid regions, with over 60 species occurring in China, and the mountainous areas of Southwest China stand out as its center of species diversity (Olmstead et al., 2001; Albach and Chase, 2004; Oxelman et al., 2005). Plants of this genus exhibit diverse morphologies, ranging from annual to perennial herbs. Some species possess multiple values in medicine, horticulture, and ecological restoration, making them ideal materials for studying angiosperm speciation, adaptive evolution, and intrafamilial systematic evolution (Tank et al., 2006; Salehi et al., 2019; Danzeng et al., 2021; Shipunov et al., 2021). Typical characteristics of this genus include: labiate corollas, terminal or axillary racemose or spicate inflorescences, compressed or globose capsules, and creeping stems in some species, which confer strong adaptability to various habitats.

In traditional taxonomy, Plantaginaceae was regarded as a small family containing only a few genera such as *Plantago* L., and was long classified independently as Plantaginales, with ambiguous phylogenetic relationships to groups like Scrophulariaceae and Gesneriaceae (Olmstead, 2002). However, in recent years, studies based on morphology by Albach et al. (2005b) and DNA markers by Olmstead et al. (2001) have shown that Plantaginaceae is the largest monophyletic clade segregated from Scrophulariaceae sensu lato. Genera such as *Veronica* L., *Plantago* L., and Rehmannia Libosch. ex Fisch. & Mey. are nested within the Plantaginaceae clade, exhibiting significant genetic divergence from Scrophulariaceae sensu stricto. Accordingly, the APG classification system (Chase et al., 2016) has completed the taxonomic revision, formally placing *Veronica* L. into Plantaginaceae and making it one of the large genera within this family (Albach et al., 2005b). However, substantial controversies remain regarding the phylogenetic relationships at different hierarchical levels (tribes, genera, and species) within the family (Hassemer et al., 2019; Mosyakin et al., 2023). Based on analyses of the internal transcribed spacer (ITS), trnL-F region, and the additional plastid *rps16* intron, Albach and Chase (2004) demonstrated that *Veronica* L. is closely related to Picrorhiza Royle ex Benth. and Veronicastrum Heist. ex Fabr., with the divergence time of the common ancestor of these three lineages dating back to approximately 10 Ma. In their study on the complete plastid genome of *Veronica arvensis* L., Liu et al. (2022) reconstructed a phylogenetic tree, which indicated that all *Veronica* species form a monophyletic clade with higher bootstrap support than those reconstructed based on plastid DNA, ribosomal DNA, and nuclear low-copy DNA. Subgenus Pentasepalae of *Veronica* L. is the most species-rich subgenus within *Veronica* in the Northern Hemisphere. Doostmohammadi et al. (2022) systematically reconstructed the phylogenetic relationships of Subgenus Pentasepalae, validated the “Iranian Plateau origin” hypothesis, and enriched the evolutionary research on *Veronica*. Based on comparative plastid genomics, Xie et al. (Xie et al., 2023) clarified that Veroniceae and Hemiphragmeae are sister groups. Zhang et al. (2025) demonstrated that *Veronica* can be further divided into two major clades, with *Veronica spicata* L. and *Veronica persica* Poir. showing a closer relationship. This research outcome provides a scientific basis for further investigating the taxonomic identification, genetic variation analysis, and phylogenetic relationships of *Veronica* species. However, insufficient species sampling, low coverage of taxa, and inadequate informative sites of molecular markers have hindered the resolution of phylogenetic trees for *Veronica* and its related genera.

In recent years, complete plastid genome sequencing has become the cornerstone of plant systematics (Neuhaus and Emes, 2000; Jansen et al., 2007; Liang et al., 2020; H-T Li et al., 2021; Song et al., 2024; Xie et al., 2025). The chloroplast genome is one of the three major DNA genomes alongside the nuclear genome and mitochondrial genome. Typically inherited maternally, it possesses a highly conserved circular DNA structure, with a size generally ranging from 115 kb to 165 kb (Dyer, 2001; Wicke et al., 2011; Daniell et al., 2016). The complete chloroplast genome exhibits a quadripartite structure, consisting of a large single copy (LSC) region, a small single copy (SSC) region, and two inverted repeat (IR) regions (Daniell et al., 2016; Yu and Dong, 2021). Differences in length are mainly attributed to the expansion/contraction of IR regions (Zhu et al., 2016)or gene loss (Magee et al., 2010). Additionally, complete chloroplast genome sequences are commonly used for phylogenetic reconstruction at lower taxonomic levels (e.g., intrageneric phylogeny) and population genetic analyses of plants. As a powerful tool for molecular phylogenetic studies (Gitzendanner et al., 2018; Shi et al., 2018), the chloroplast genome has been widely applied in Plantaginaceae plants as well as other Lamiales taxa (Rønsted et al., 2002; Liu et al., 2021; Mower et al., 2021; Xie et al., 2023). Previous studies on *Veronica* classification employed one to several molecular markers, such as ITS, trnL-F, and psbA-trnH sequences, while some studies only used ITS sequences, leaving many phylogenetic and taxonomic questions unresolved. Additionally, the lack of complete chloroplast genome sequences has severely hindered the evaluation and analysis of genetic diversity in *Veronica* germplasm resources. Molecular clocks can estimate species divergence times and reconstruct evolutionary histories through the mutation rates of gene sequences (Drummond and Rambaut, 2007; Dos Reis et al., 2014). Based on molecular clock analysis of chloroplast genomes from 34 Veronica-related taxa and 2 outgroups, Hai et al. (2024) demonstrated that the ancestral lineage of *Veronica* originated approximately 12.76 Ma, initiated initial diversification at 9.9 Ma, and further validated that the core divergence time of *Veronica* is approximately 9.09 Ma.

In the present study, three species of *Veronica* L. were used as research materials to explore plastome variation and phylogenetic relationships within *Veronica* and its related Plantaginaceae lineages. We report three newly assembled chloroplast genomes of *Veronica ciliata* Fisch., *Veronica biloba* L., and *Veronica vandellioides* Maxim. By generating three complete chloroplast genomes of *Veronica*, we aimed to: (1) systematically characterize core plastomic features of *Veronica*, including genome architecture, repetitive sequences, and IR/SC boundary variations, through comprehensive comparative genomic analyses, and identify plastid mutation hotspots to supply molecular markers for species discrimination and genetic diversity evaluation; (2) reconstruct phylogenetic relationships among *Veronica* and its closely related genera and resolve the internal phylogenetic backbone of the genus; (3) estimate lineage divergence times of *Veronica* and perform ancestral state reconstruction to help interpret the evolutionary origins of the genus. These analyses provide plastome resources for species identification and future comparative studies, while recognizing that plastid data alone cannot fully represent species histories affected by hybridization, introgression, or incomplete lineage sorting.

## 2 Materials and methods

### 2.1 Sampling, extraction, and genome sequencing

Fresh leaves of the three *Veronica* species were collected from natural populations (**Table 1**). Taxonomic authentication of the plant materials was conducted by Dr. Zhixi Fu, and the corresponding voucher specimens were deposited in the Herbarium of Sichuan Normal University (SCNU)(contact person: Dr. Zhixi Fu, fuzx2017@sicnu.edu.cn). Since the studied species are not listed in the National Key Protected Wild Plants of China, no collection permit was required. Genomic DNA was isolated from the leaf tissues following a CTAB-based protocol (Allen et al., 2006; Doyle and Doyle, 1987) using the Plant Genomic DNA Extraction Kit (Tiangen, Beijing, China). DNA libraries were constructed with the Illumina Paired-End DNA Library Kit (Illumina Inc., San Diego, CA, USA), and high-throughput sequencing was performed on an Illumina Hiseq 2000 platform. The raw sequencing data generated for each of the three species comprised 150-bp paired-end reads. Primary and secondary quality control procedures were subsequently performed on the raw data to obtain clean data. Complete chloroplast genome sequences downloaded from NCBI were used as comparative references (**Table 1**), and species names in the final dataset were checked against Flora of China and the original GenBank records.

**Table 1:** Basic information of the 93 plant species utilized in this study.

taxa	Species	GenBank	Plastome size (bp)	Voucher No.	Locality information of newly sequenced species
*Adenosma*	*Adenosma bracteosa*	OR367324.1	154,106	/	/
	*Adenosma glutinosum*	NC_072589.1	154,220	/	/
*Angelonia*	*Angelonia angustifolia*	NC_061393.1	154,316	/	/
*Antirrhinum*	*Antirrhinum hispanicum*	NC_068045.1	152,481	/	/
	*Antirrhinum majus* var. *pseudomajus*	OL977690.1	152,619	/	/
	*Antirrhinum majus*	PP316519.1	152,621	/	/
*Bacopa*	*Bacopa monnieri*	NC_047469.1	152,495	/	/
*Callitriche*	*Callitriche cophocarpa*	NC_080351.1	150,324	/	/
	*Callitriche hermaphroditica*	NC_080352.1	150,042	/	/
	*Callitriche palustris*	NC_080353.1	150,071	/	/
	*Callitriche stagnalis*	NC_066447.1	150,879	/	/
*Chaenorhinum*	*Chaenorhinum minus subsp. minus*	PP328793.1	151,423	/	/
*Cymbalaria*	*Cymbalaria muralis subsp. muralis*	PP335047.1	152,665	/	/
*Deinostema*	*Deinostema violacea*	NC_072590.1	154,280	/	/
*Digitalis*	*Digitalis grandiflora*	PP335057.1	153,115	/	/
	*Digitalis purpurea*	NC_068046.1	152,999	/	/
*Ellisiophyllum*	*Ellisiophyllum pinnatum*	NC_072592.1	152,424	/	/
*Globularia*	*Globularia bisnagarica*	PP336757.1	152,024	/	/
	*Globularia cordifolia*	PP336755.1	152,818	/	/
	*Globularia nudicaulis*	PP336756.1	151,346	/	/
*Hemiphragma*	*Hemiphragma heterophyllum*	NC_045398.1	152,700	/	/
*Hippuris*	*Hippuris montana*	NC_072276.1	152,375	/	/
	*Hippuris vulgaris*	NC_054196.1	152,698	/	/
*Lagotis*	*Lagotis brachystachya*	PP234541.1	152,610	/	/
	*Lagotis brevituba*	NC_057062.1	152,967	/	/
	*Lagotis yunnanensis*	NC_056954.1	152,789	/	/
*Limnophila*	*Limnophila aromatica*	PV890005.1	152,121	/	/
	*Limnophila rugosa*	PV890006.1	154,041	/	/
	*Limnophila sessiliflora*	NC_063679.1	152,395	/	/
*Linaria*	*Linaria angustissima*	PP336775.1	150,621	/	/
	*Linaria buriatica*	NC_072593.1	150,665	/	/
	*Linaria japonica*	NC_085416.1	150,660	/	/
	*Linaria vulgaris*	NC_068047.1	150,708	/	/
*Littorella*	*Littorella uniflora*	NC_068048.1	130,759	/	/
*Neopicrorhiza*	*Neopicrorhiza scrophulariiflora*	NC_057075.1	152,642	/	/
*Penstemon*	*Penstemon cyaneus*	NC_061962.1	152,604	/	/
	*Penstemon digitalis*	NC_086665.1	152,993	/	/
	*Penstemon personatus*	NC_061960.1	152,602	/	/
	*Penstemon rostriflorus*	NC_061961.1	152,598	/	/
*Plantago*	*Plantago arborescens*	NC_088462.1	149,298	/	/
	*Plantago aristata*	NC_088463.1	149,887	/	/
	*Plantago erecta*	NC_088468.1	149,381	/	/
	*Plantago lagocephala*	NC_088472.1	149,672	/	/
	*Plantago lagopus*	NC_041420.1	145,936	/	/
	*Plantago lanceolata*	NC_068049.1	149,743	/	/
	*Plantago minuta*	NC_088474.1	149,658	/	/
	*Plantago ovata*	NC_041421.1	149,739	/	/
*Russelia*	*Russelia equisetiformis*	NC_072594.1	153,337	/	/
*Schweinfurthia*	*Schweinfurthia imbricata*	PV137603.1	153,206	/	/
	*Schweinfurthia papilionacea*	PV097193.1	153,238	/	/
*Scoparia*	*Scoparia dulcis*	MZ242235.1	153,701	/	/
*Veronica*	*Veronica agrestis*	NC_068050.1	150,197	/	/
	*Veronica anagallis-aquatica*	NC_082107.1	151,277	/	/
	*Veronica aphylla subsp. aphylla*	PP622714.1	151,110	/	/
	*Veronica arvensis*	NC_064998.1	149,386	/	/
	*Veronica beccabunga*	PP622716.1	150,512	/	/
	*Veronica bellidioides*	PP622717.1	150,820	/	/
	*Veronica biloba*	PX776077.1	150,202	SDX0410	Seda County, Sichuan Province
	*Veronica chamaedrys*	NC_068051.1	150,562	/	/
	*Veronica chamaedrys subsp. chamaedry*	PP622718.1	150,625	/	/
	*Veronica ciliata*	PX776076.1	151,098	SDX0431	Seda County, Sichuan Province
	*Veronica eriogyne*	NC_058571.1	151,083	/	/
	*Veronica fruticans*	PP622719.1	151,178	/	/
	*Veronica hederifolia*	PP840335.1	149,508	/	/
	*Veronica nakaiana*	MT422349.1	152,319	/	/
	*Veronica peregrina*	NC_077523.1	151,249	/	/
	*Veronica persica*	PP840336.1	150,282	/	/
	*Veronica polita*	NC_060687.1	150,191	/	/
	*Veronica prostrata*	PP622721.1	150,614	/	/
	*Veronica serpyllifolia*	PP622722.1	150,982	/	/
	*Veronica sieboldiana*	PX870807.1	152,262	/	/
	*Veronica spicata*	NC_084407.1	152,225	/	/
	*Veronica undulata*	NC_077524.1	151,198	/	/
	*Veronica urticifolia*	PP622723.1	150,888	/	/
	*Veronica vandellioides*	PX776078.1	151,159	SDX0039	Seda County, Sichuan Province
	*Veronica officinalis*	PP840337.1	150,916	/	/
*Veronicastrum*	*Veronicastrum axillare*	NC_056895.1	152,691	/	/
	*Veronicastrum brunonianum*	NC_082033.1	152,587	/	/
	*Veronicastrum latifolium*	NC_069913.1	152,966	/	/
	*Veronicastrum rhombifolium*	NC_082029.1	152,693	/	/
	*Veronicastrum robustum*	NC_082030.1	152,658	/	/
	*Veronicastrum stenostachyum*	NC_082031.1	152,957	/	/
	*Veronicastrum yunnanense*	NC_082032.1	152,779	/	/
outgroup	*Verbascum songaricum*	NC_085506.1	153,291	/	/
	*Verbascum chaixii*	NC_085505.1	153495	/	/
	*Coffea arabica*	NC_008535.1	155,189	/	/
	*Solanum lycopersicum*	NC_007898.3	155,461	/	/
	*Jasminum nudiflorum*	NC_008407.1	165,121	/	/
	*Avicennia marina*	MT108381.1	147,909	/	/
	*Acanthus ilicifolius*	MW174172.1	150,758	/	/
	*Salvia miltiorrhiza*	NC_020431.1	151,394	/	/
	*Sesamum indicum*	NC_016433.1	153,324	/	/
	*Forsythia suspensa*	MF579702.1	156,404	/	/

### 2.2 Assembly and annotation of chloroplast genome

High-quality sequencing reads from *V. ciliata*, *V. biloba* and *V. vandellioides* were aligned to the reference sequence of *Veronica agrestis* L. using Bowtie2 v.2.4.5-Linux (Langmead et al., 2019). SAMtools v.1.15-Linux (Danecek et al., 2021) was then used to filter raw data. Only reads successfully mapped to the reference sequence were retained for subsequent plastome assembly. For chloroplast genome assembly, clean paired-end reads were assembled with SPAdes 3.15.1 using default parameters. Assembly graphs were inspected with Bandage to confirm circular plastome structure and to remove ambiguous contigs (Wick et al., 2015; Zhao et al., 2020). To evaluate assembly quality, reads were mapped back to each assembled plastome using BWA v0.7.17, and sequencing depth and coverage were calculated across the genome. The LSC, SSC and IR boundaries were checked by combining read-depth evidence, the Bandage assembled graph, and manual inspection in Geneious Prime. The IR boundaries of the three species were verified using the -IR module of the Linux version of CPStools (Huang et al., 2024). The resulting assemblies were initially annotated using PGA and then annotated with CPGAVAS2 (Shi et al., 2019) (http://47.96.249.172:16019/analyzer/home). Preliminary GenBank-format files were checked with CPStools (Huang et al., 2024), and annotation errors were manually corrected in Geneious Prime. Circular plastome maps were generated with Chloroplot software (Zheng et al., 2020), and general plastome features were summarized in Geneious R11.

### 2.3 Comparative genome analysis

IRscope (https://irscope.shinyapps.io/irapp/) was employed to visualize and compare the junctions among the LSC, IR, and SSC regions of the chloroplast genomes (Amiryousefi et al., 2018). With the *V. biloba* as reference, the homology of these sequences was visualized using the mVISTA program (Frazer et al., 2004) with the LAGAN mode (Brudno et al., 2003).

### 2.4 Repeat sequences and simple sequence repeats analysis

In this study, REPuter (Kurtz et al., 2001) was employed to identify interspersed repeat elements, including direct (forward), inverted (palindromic), complement, and reverse repeats. Parameters were set to a Hamming distance of 3, a minimum repeat length of 30 bp, and a maximum computed repeat length of 50 bp. Simple sequence repeats (SSRs) in the complete chloroplast genomes were detected using the MISA (Beier et al., 2017), with thresholds of 10, 5, 4, 3, 3, and 3 repeat units for mono-, di-, tri-, tetra-, penta- and hexanucleotide SSRs, respectively.

For nucleotide diversity analysis, complete chloroplast genome sequences from the three *Veronica* species were aligned using the MAFFT “–auto” strategy. The three-species dataset was used for intra-generic comparative analyses because complete plastomes were available for these taxa and could be aligned confidently across coding and non-coding regions. Nucleotide diversity (Pi) of chloroplast genomes was calculated via the online platform GenePioneer (http://cloud.genepioneer.com:9929/#/tool/alltool/detail/328), with a window length of 600 bp and a step size of 200 bp. Regions with Pi values greater than three times the genome-wide mean were treated as highly variable regions.

### 2.5 Codon usage analysis

Synonymous codon usage and relative synonymous codon usage (RSCU) of the chloroplast genomes of *Veronica* were analyzed using CPStools software (Huang et al., 2024). An RSCU value greater than 1 indicates a higher usage frequency of the corresponding codon, while a value less than 1 denotes a lower usage frequency. Codons with no usage bias are assigned an RSCU value of 1.00.

### 2.6 Phylogenetic analysis

A dataset was compiled using complete chloroplast genomes from 93 plant species, comprising 83 taxa of the Plantaginaceae and 10 outgroups (**Table 1**). Phylogenetic analyses were performed with the maximum likelihood approach implemented in IQ-TREE. All corresponding chloroplast genome sequences were retrieved and downloaded from the GenBank database of the National Center for Biotechnology Information (NCBI)(https://www.ncbi.nlm.nih.gov/Genbank). Multiple sequence alignment was performed using MAFFT v7.526 (Katoh and Standley, 2013; K Li et al., 2021), and the sequences were trimmed with CPStools (Huang et al., 2024). Maximum likelihood (ML) tree construction was performed in IQ-TREE under the GTRGAMMA model with 1000 bootstrap replicates (Nguyen et al., 2015). The complete taxon list, GenBank accessions and taxonomic assignments are provided in **Table 1**.

### 2.7 Morphological trait analysis

Six key morphological characters (inflorescence type, corolla color, style morphology, calyx indumentum, sepal equality and stamen number) were selected for ancestral state reconstruction in this study. These characters are taxonomically diagnostic and provide critical references for phylogenetic analyses of *Veronica*. Inflorescence morphology, calyx structure and stamen number are genetically conserved with clear character discontinuities among clades, which can be used to distinguish closely related species and higher-level lineages. Corolla color, style morphology and calyx indumentum are tightly linked to pollination adaptation, reproductive strategies and habitat stress tolerance and these traits carry abundant ecological and evolutionary information. All characters were coded based on records from *Flora of China* (https://www.iplant.cn/foc). These morphological traits showed high observational stability and complete datasets. Ancestral state reconstruction was performed by integrating the morphological matrix with the phylogenetic framework constructed from chloroplast genomes. The evolutionary trajectories of floral characters within *Veronica* can be assessed using this combined approach. This approach helps compensate for the limitation that molecular sequences alone reflect genetic divergence but do not reconstruct phenotypic evolutionary histories. This therefore provides morphological evolutionary evidence to support subsequent taxonomic revisions of infrageneric taxa and inferences on the drivers of species differentiation. Coding schemes for the corresponding character states were defined as follows (**Supplementary Table 2**): (A) Inflorescence: (0) terminal raceme, (1) lateral raceme, (2) spike, (3) capitulum, (4) solitary axillary flower, (5) thyrse; (B) Corolla color: (0) white, (1) pale yellow, (2) bluish-purple, (3) others; (C) Style type: (0) filiform and exserted, (1) long-styled; (D) Calyx indumentum: (0) glabrous, (1) pubescent; (E) Sepal subequability: (0) equal, (1) unequal; (F) Stamen number: (0) four, (1) two, (2) one, (3) five. The ancestral states of *Veronica* and its relatives were reconstructed in Mesquite v.4.01 (https://www.mesquiteproject.org) based on the recovered phylogenetic tree topology. Analyses were performed via the “Character History Source” function and the “Likelihood Ancestral States” method under the one-parameter Markov k-state probability (Mk1) model (Lewis, 2001).

### 2.8 Divergence time estimations

Species divergence times were estimated using two complementary Bayesian frameworks to cross-validate the results. BEAST v.1.10.4 (Drummond et al., 2012) was used for the primary analysis. Divergence times were inferred from complete chloroplast genome data under the uncorrelated-lognormal relaxed clock model. All phylogenetic calibrations were secondary calibrations referenced to Surina et al. (2014) and Meudt et al (Meudt et al., 2015), whose age constraints were supported by fossil paleontological and geomorphological data. The crown node of *Veronica* was fixed at 14.9 Ma. The Yule model was selected as the tree prior. The Markov Chain Monte Carlo (MCMC) analysis was run for 10 million generations, with sampling conducted every 1000 generations. The first 10% of samples were removed as burn-in. Convergence and posterior distributions were validated in Tracer v.1.7.2, with all effective sample size (ESS) values exceeding 200. TreeAnnotator v.1.6 was used to summarize the BEAST output, generate the maximum clade credibility tree, and calculate 95% highest posterior density (HPD) intervals for all key nodes. As an independent cross-check, a parallel divergence time analysis was performed using the MCMCtree module in PAML v.4.9. The resulting time tree from the MCMCtree pipeline was visualized using its built-in plotting functions to confirm the consistency of divergence time estimates.

## 3 Results

### 3.1 General features of the chloroplast genomes of *Veronica* (Plantaginaceae)

**Figure 1:**
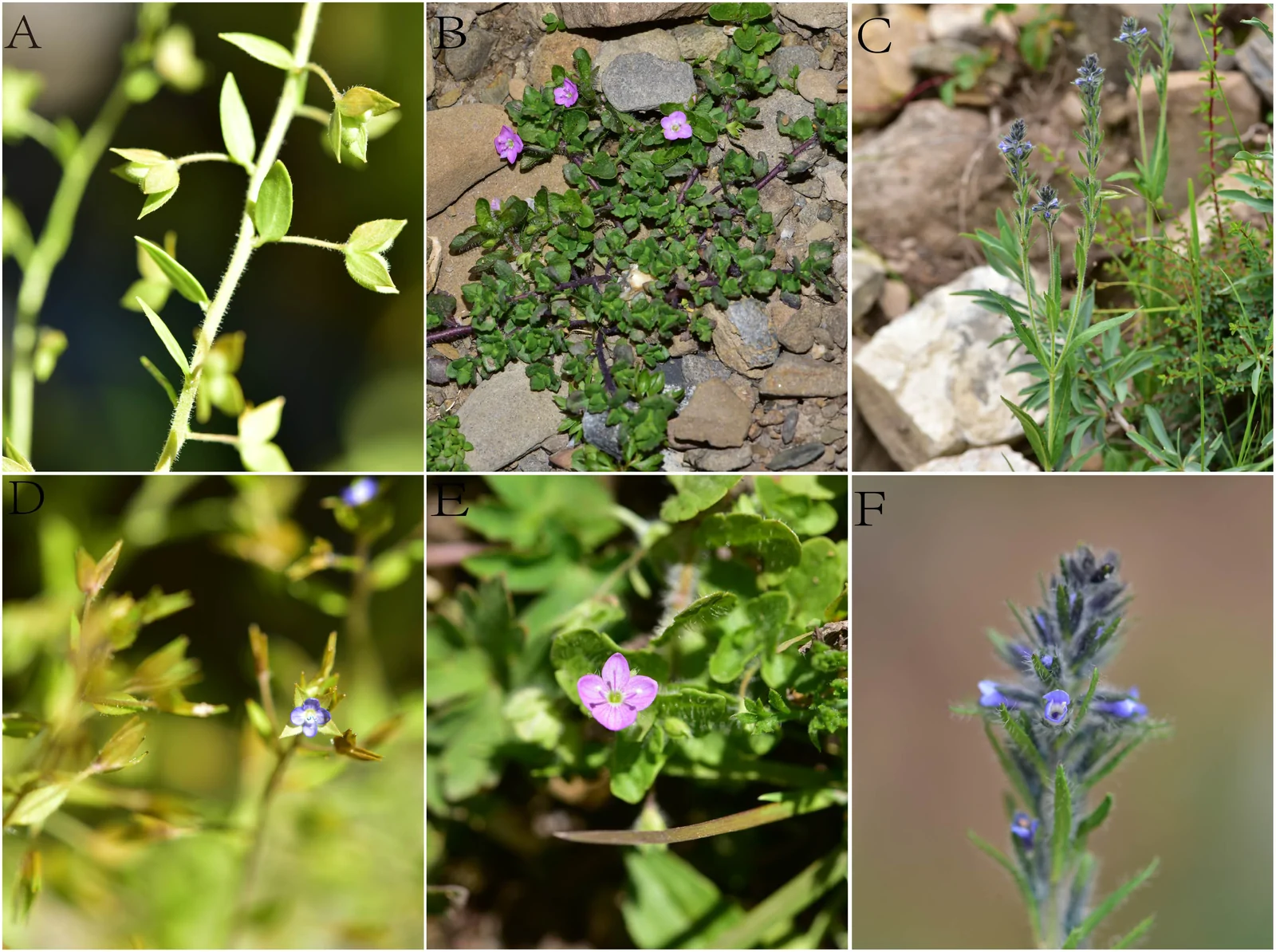
Photographs of three newly sequenced *Veronica* species. **(A)** Overall view of a leaf of *Veronica biloba*. **(B)** Overall view of a *Veronica vandellioides* plant. **(C)** Overall view of a *Veronica ciliata* plant. **(D)** Detail of a flower of *Veronica biloba*. **(E)** Detail of a flower of *Veronica vandellioides*. **(F)** Detail of a flower of *Veronica ciliata*.

The complete chloroplast genomes of three *Veronica* species, including *V. biloba*, *V. ciliata* and *V. vandellioides*, were obtained in this study (**Figure 1**). The sequencing-depth maps showed continuous coverage across the plastomes without obvious gaps or extreme depth fluctuations, supporting the reliability of the assemblies for subsequent comparative analysis.

**Figure 2:**
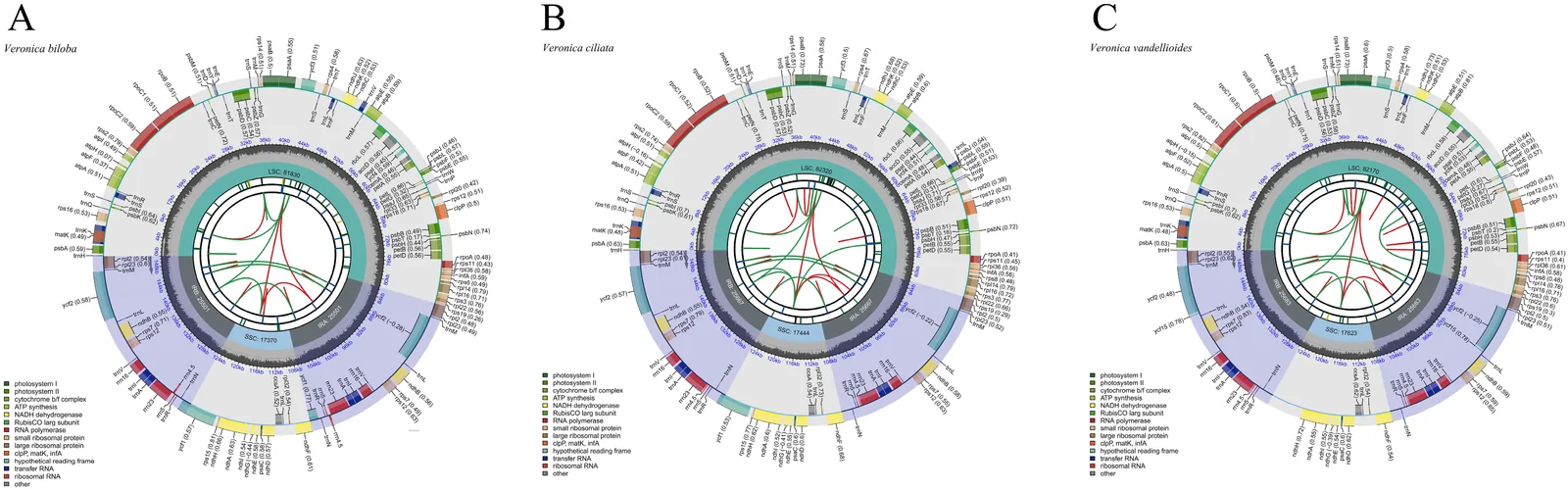
**(A)** Circular map of the chloroplast genome of *Veronica biloba*. The map of the complete cp genome was generated using Chloroplot. The outermost circle shows the distribution of genes on the positive (outer) and reverse (inner) strands, with functional categories color-coded. The second circle displays GC content variation (dark gray = high GC content, light gray = low AT content). The inner structure illustrates the typical quadripartite organization composed of the large single-copy (LSC) region, small single-copy (SSC) region, and two inverted repeat (IR) regions. Gene positions and orientations are indicated by arrows. **(B)** Circular map of the chloroplast genome of *Veronica ciliata*. The map of the complete cp genome was generated using Chloroplot. The outermost circle shows the distribution of genes on the positive (outer) and reverse (inner) strands, with functional categories color-coded. The second circle displays GC content variation (dark gray = high GC content, light gray = low AT content). The inner structure illustrates the typical quadripartite organization composed of the large single-copy (LSC) region, small single-copy (SSC) region, and two inverted repeat (IR) regions. Gene positions and orientations are indicated by arrows. **(C)** Circular map of the chloroplast genome of *Veronica vandellioides*. The map of the complete cp genome was generated using Chloroplot. The outermost circle shows the distribution of genes on the positive (outer) and reverse (inner) strands, with functional categories color-coded. The second circle displays GC content variation (dark gray = high GC content, light gray = low AT content). The inner structure illustrates the typical quadripartite organization composed of the large single-copy (LSC) region, small single-copy (SSC) region, and two inverted repeat (IR) regions. Gene positions and orientations are indicated by arrows.

**Figure 3:**
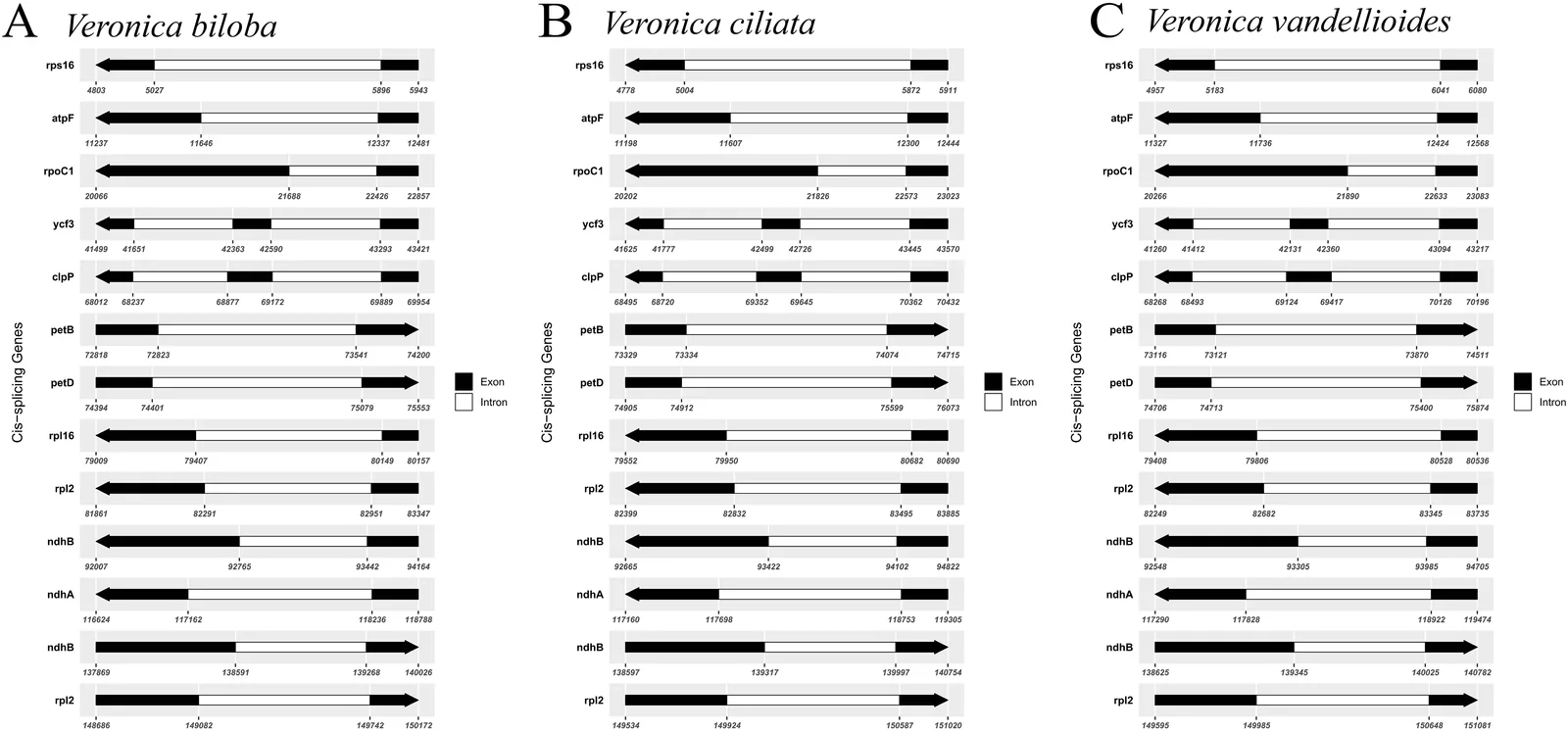
**(A)** Schematic map of the cis-splicing genes in the cp genome of *Veronica biloba*. Exon–intron organization of representative genes is shown with black boxes representing exons and connecting lines representing introns. **(B)** Schematic map of the cis-splicing genes in the cp genome of *Veronica ciliata*. Exon–intron organization of representative genes is shown with black boxes representing exons and connecting lines representing introns. **(C)** Schematic map of the cis-splicing genes in the cp genome of *Veronica vandellioides*. Exon–intron organization of representative genes is shown with black boxes representing exons and connecting lines representing introns.

**Figure 4:**
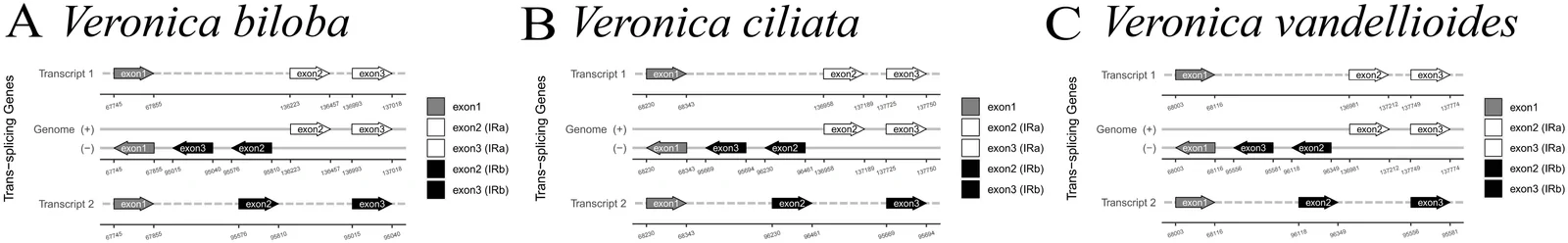
**(A)** Schematic map of the trans-splicing genes in the cp genome of *Veronica biloba*. **(B)** Schematic map of the trans-splicing genes in the cp genome of *Veronica ciliata*. **(C)** Schematic map of the trans-splicing genes in the cp genome of *Veronica vandellioides.*.

All three genomes were circular double-stranded DNA molecules and exhibited the typical quadripartite structure of angiosperm plastomes, consisting of a large single-copy (LSC) region, a small single-copy (SSC) region, and a pair of inverted repeat (IRa/IRb) regions. The plastome lengths were 151,159 bp for *V. ciliata*, 151,098 bp for *V. biloba* and 151,159 bp for *V. vandellioides* (**Table 2**), with GC content ranging from 37.9% to 38.2%. A total of 130–132 unique genes were annotated, including 86–87 protein-coding genes, 36–37 tRNA genes, and 8 rRNA genes. The three plastomes shared three genes with two introns, namely *rps12*, *clpP* and *ycf3* (**Table 3**). *V. biloba* and *V. ciliata* each contained 15 single-intron genes, whereas *V. vandellioides* contained 14 single-intron genes (**Figures 2**–**4**).

**Table 2:** Comparative analysis of chloroplast genomes of three *Veronica* species.

Species	*Veronica ciliata*	*Veronica biloba*	*Veronica vandellioides*
GenBank accession	PX776076.1	PX776077.1	PX776078.1
SRA accession numbers	SRR39066514	SRR39066513	SRR39066512
Plastome size (bp)	151,098	150,202	151,159
LSC length (bp)	82,320	81,830	82,170
IR length (bp)	51,334	51,002	51,366
SSC length (bp)	17,444	17,370	17,623
GC content (%)	38.00%	37.90%	38.20%
Number of genes	131	132	130
Protein coding genes	86	87	86
tRNA genes	37	37	36
rRNA genes	8	8	8

**Table 3:** List of genes found in the complete chloroplast genomes of the three *Veronica* species.

Species	Category	Gene group	Gene name
*Veronica biloba*	Photosynthesis	Subunits of photosystem I	*psaB, psaA, psaI, psaJ, psaC*
		Subunits of photosystem II	*psbA, psbK, psbI, psbM, psbD, psbC, psbZ, psbJ, psbL, psbF, psbE, psbB, psbT, psbN, psbH*
		Subunits of NADH dehydrogenase	ndhJ, ndhK, ndhC, ndhB(2)*, ndhF, ndhD, ndhE, ndhG, ndhI, ndhA*, ndhH
		Subunits of cytochrome b/f complex	petN, petA, petL, petG, petB*, petD*
		Large subunit of rubisco	*rbcL*
		Subunits of ATP synthase	atpA, atpF*, atpH, atpI, atpE, atpB
	Self-replication	Proteins of large ribosomal subunit	rpl33, rpl20, rpl36, rpl14, rpl16*, rpl22, rpl2(2)*, rpl23(2), rpl32
		Proteins of small ribosomal subunit	rps12(2)**, rps16*, rps2, rps14, rps4, rps18, rps11, rps8, rps3, rps19, rps7(2), rps15
		Subunits of RNA polymerase	rpoC2, rpoC1*, rpoB, rpoA
		Ribosomal RNAs	*rrn16S(2), rrn23S(2), rrn4.5S(2), rrn5S(2)*
		Transfer RNAs	trnH-GUG, trnK-UUU*, trnQ-UUG, trnS-GCU, trnS-CGA*, trnR-UCU, trnC-GCA, trnD-GUC, trnY-GUA, trnE-UUC, trnT-GGU, trnS-UGA, trnG-GCC, trnM-CAU(4), trnS-GGA, trnT-UGU, trnL-UAA*, trnF-GAA, trnV-UAC*, trnW-CCA, trnP-UGG, trnL-CAA(2), trnV-GAC(2), trnI(2)*, trnA-UGC(2)*, trnR-ACG(2), trnN-GUU(2), trnL-UAG
	Other genes	Maturase	*matK*
		Protease	clpP**
		Envelope membrane protein	*cemA*
		Acetyl-CoA carboxylase	*accD*
		c-type cytochrome synthesis gene	*ccsA*
		Translation initiation factor	*infA*
		Conserved open reading frames	ycf3**, ycf4, ycf2(2), ycf1(2)
*Veronica ciliata*	Photosynthesis	Subunits of photosystem I	*psaB, psaA, psaI, psaJ, psaC*
		Subunits of photosystem II	*psbA, psbK, psbI, psbM, psbD, psbC, psbZ, psbJ, psbL, psbF, psbE, psbB, psbT, psbN, psbH*
		Subunits of NADH dehydrogenase	ndhJ, ndhK, ndhC, ndhB(2)*, ndhF, ndhD, ndhE, ndhG, ndhI, ndhA*, ndhH
		Subunits of cytochrome b/f complex	petN, petA, petL, petG, petB*, petD*
		Large subunit of rubisco	*rbcL*
		Subunits of ATP synthase	atpA, atpF*, atpH, atpI, atpE, atpB
	Self-replication	Proteins of large ribosomal subunit	rpl33, rpl20, rpl36, rpl14, rpl16*, rpl22, rpl2(2)*, rpl23(2), rpl32
		Proteins of small ribosomal subunit	rps12(2)**, rps16*, rps2, rps14, rps4, rps18, rps11, rps8, rps3, rps19, rps7(2), rps15
		Subunits of RNA polymerase	rpoC2, rpoC1*, rpoB, rpoA
		Ribosomal RNAs	*rrn16S(2), rrn23S(2), rrn4.5S(2), rrn5S(2)*
		Transfer RNAs	trnH-GUG, trnK-UUU*, trnQ-UUG, trnS-GCU, trnS-CGA*, trnR-UCU, trnC-GCA, trnD-GUC, trnY-GUA, trnE-UUC, trnT-GGU, trnS-UGA, trnG-GCC, trnM-CAU(4), trnS-GGA, trnT-UGU, trnL-UAA*, trnF-GAA, trnL-UAG(2)*, trnW-CCA, trnP-UGG, trnL-CAA(2), trnV-GAC(2), trnI(2)*, trnA-UGC(2)*, trnR-ACG(2), trnN-GUU(2)
	Other genes	Maturase	*matK*
		Protease	clpP**
		Envelope membrane protein	*cemA*
		Acetyl-CoA carboxylase	*accD*
		c-type cytochrome synthesis gene	*ccsA*
		Translation initiation factor	*infA*
		Conserved open reading frames	ycf3**, ycf4, ycf2(2), ycf1
*Veronica vandellioides*	Photosynthesis	Subunits of photosystem I	*psaB, psaA, psaI, psaJ, psaC*
		Subunits of photosystem II	*psbA, psbK, psbI, psbM, psbD, psbC, psbZ, psbJ, psbL, psbF, psbE, psbB, psbT, psbN, psbH*
		Subunits of NADH dehydrogenase	ndhJ, ndhK, ndhC, ndhB(2)*, ndhF, ndhD, ndhE, ndhG, ndhI, ndhA*, ndhH
		Subunits of cytochrome b/f complex	petN, petA, petL, petG, petB*, petD*
		Large subunit of rubisco	*rbcL*
		Subunits of ATP synthase	atpA, atpF*, atpH, atpI, atpE, atpB
	Self-replication	Proteins of large ribosomal subunit	rpl33, rpl20, rpl36, rpl14, rpl16*, rpl22, rpl2(2)*, rpl23(2), rpl32
		Proteins of small ribosomal subunit	rps12(2)**, rps16*, rps2, rps14, rps4, rps18, rps11, rps8, rps3, rps19, rps7(2)
		Subunits of RNA polymerase	rpoC2, rpoC1*, rpoB, rpoA
		Ribosomal RNAs	*rrn16S(2), rrn23S(2), rrn4.5S(2), rrn5S(2)*
		Transfer RNAs	trnH-GUG, trnK-UUU*, trnQ-UUG, trnS-GCU, trnS-CGA*, trnR-UCU, trnC-GCA, trnD-GUC, trnY-GUA, trnE-UUC, trnT-GGU, trnS-UGA, trnG-GCC, trnM-CAU(4), trnS-GGA, trnT-UGU, trnL-UAA*, trnF-GAA, trnW-CCA, trnP-UGG, trnL-CAA(2), trnV-GAC(2), trnI(2)*, trnA-UGC(2)*, trnR-ACG(2), trnN-GUU(2), trnL-UAG
	Other genes	Maturase	*matK*
		Protease	clpP**
		Envelope membrane protein	*cemA*
		Acetyl-CoA carboxylase	*accD*
		c-type cytochrome synthesis gene	*ccsA*
		Translation initiation factor	*infA*
		Conserved open reading frames	ycf3**, ycf4, ycf2(2), ycf15(2)

Gene*, gene with one introns, Gene**, gene with two introns, Gene(2), number of copies of multi-copy genes.

### 3.2 Analysis of repetitive sequences

**Figure 5:**
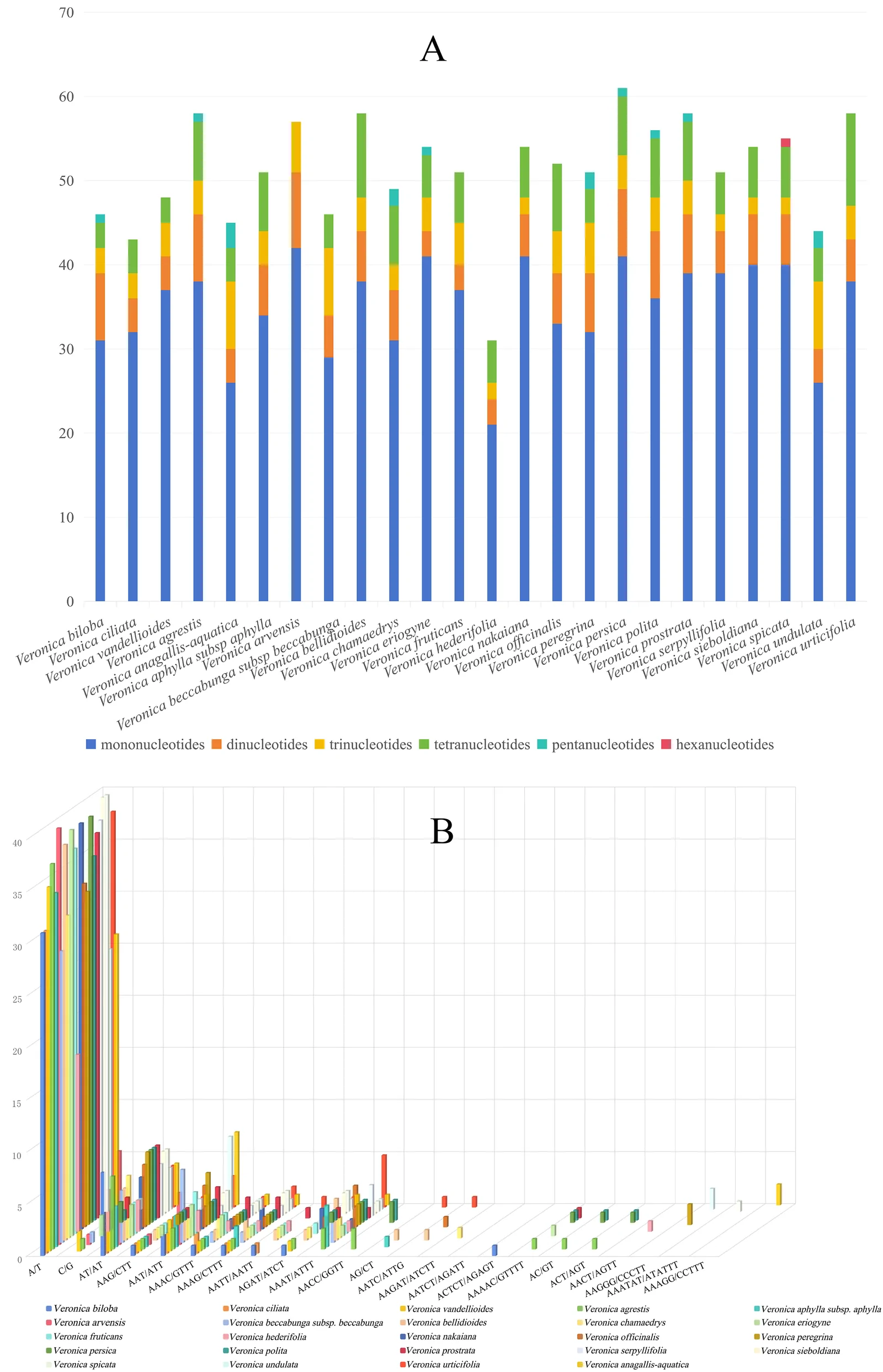
Comparison of simple sequence repeats (SSRs) among 24 *Veronica* chloroplast genomes. **(A)** Number of SSR loci detected in each plastome. **(B)** Distribution of SSR motif types among the 24 *Veronica* plastomes.

SSR variation was compared across 24 *Veronica* plastomes, including the three newly sequenced species and 21 publicly available plastomes (**Figure 5A**). The number of SSR loci ranged from 31 in *Veronica hederifolia* to 61 in *Veronica persica*, indicating moderate interspecific variation in SSR abundance rather than complete numerical similarity. Nevertheless, motif composition was broadly conserved across the genus, with A/T mononucleotide repeats and other AT-rich motifs forming the dominant SSR types. Pentanucleotide repeats were detected only in *V. biloba* among the sampled taxa, and the C/G motif was detected only in *V. vandellioides* (**Figure 5B**).

**Figure 6:**
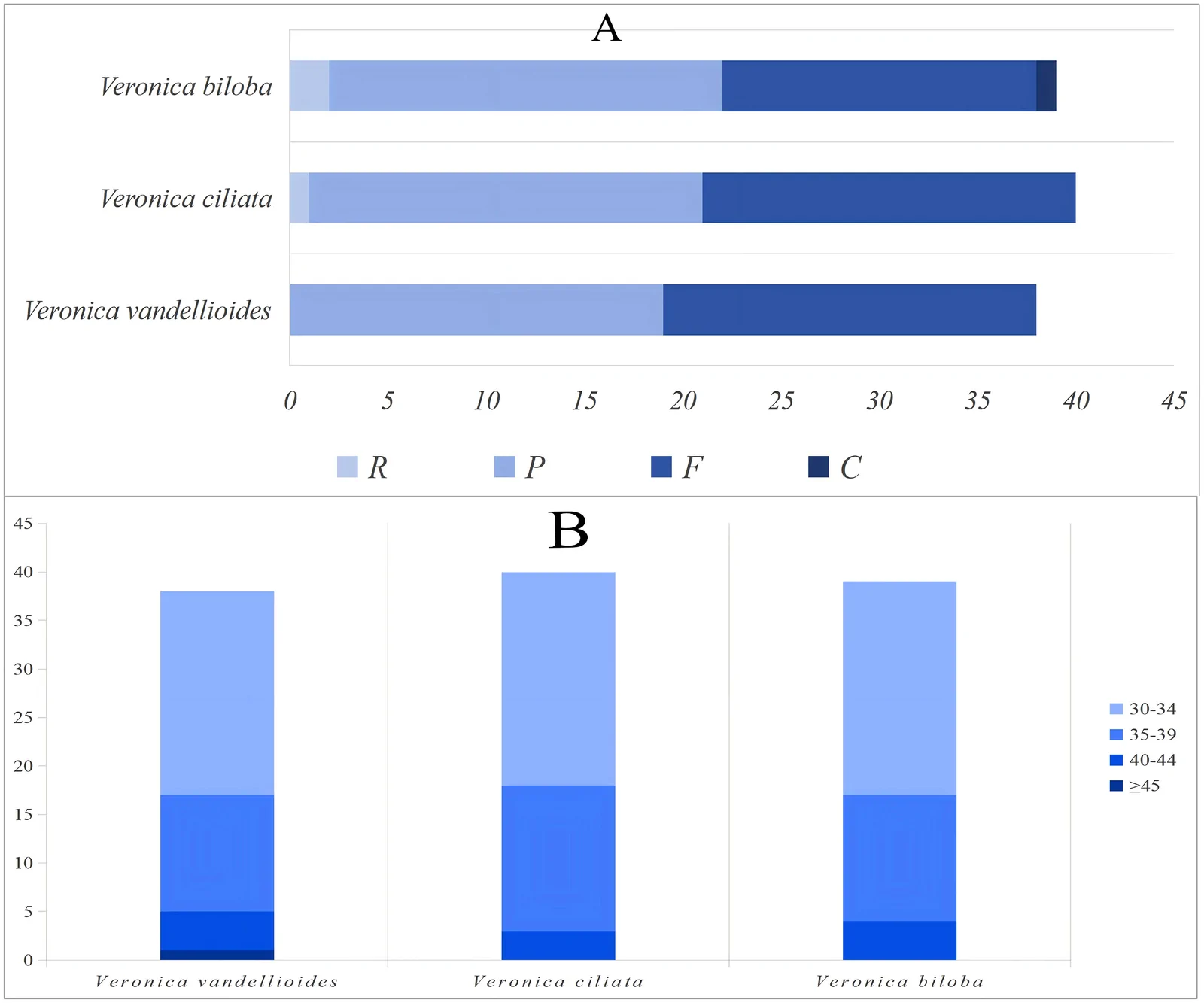
Number and length distribution of long repeat sequences identified from the three newly sequenced *Veronica* chloroplast genomes. Repeats were detected with a Hamming distance of 3 and a minimum repeat length of 30 bp. **(A)** Number of forward (F), palindromic (P), reverse (R) and complement (C) repeats. **(B)** Length distribution of long repeat sequences.

Forward, palindromic, complement and reverse repeats were detected in the three newly sequenced chloroplast genomes (**Figure 6A**). Among them, *V. biloba* possessed all four types of repeats. *V. ciliata* possessed forward, palindromic, and reverse repeats, but no complement repeats were detected. *V. vandellioides* possessed only palindromic and reverse repeats. On average, approximately 38–39 repeat sequences were identified in these genomes, including 1–2 forward repeats, 19–20 palindromic repeats, 16–19 reverse repeats, and one complement repeat. However, the complement repeat was only detected in *V. biloba*, with a single copy. Furthermore, repeat sequences of 30–34 bp in length were the most common type in these genomes (**Figure 6B**), whereas repeats longer than 45 bp were only detected in *V. vandellioides*.

### 3.3 Boundary region expansions and contractions

**Figure 7:**
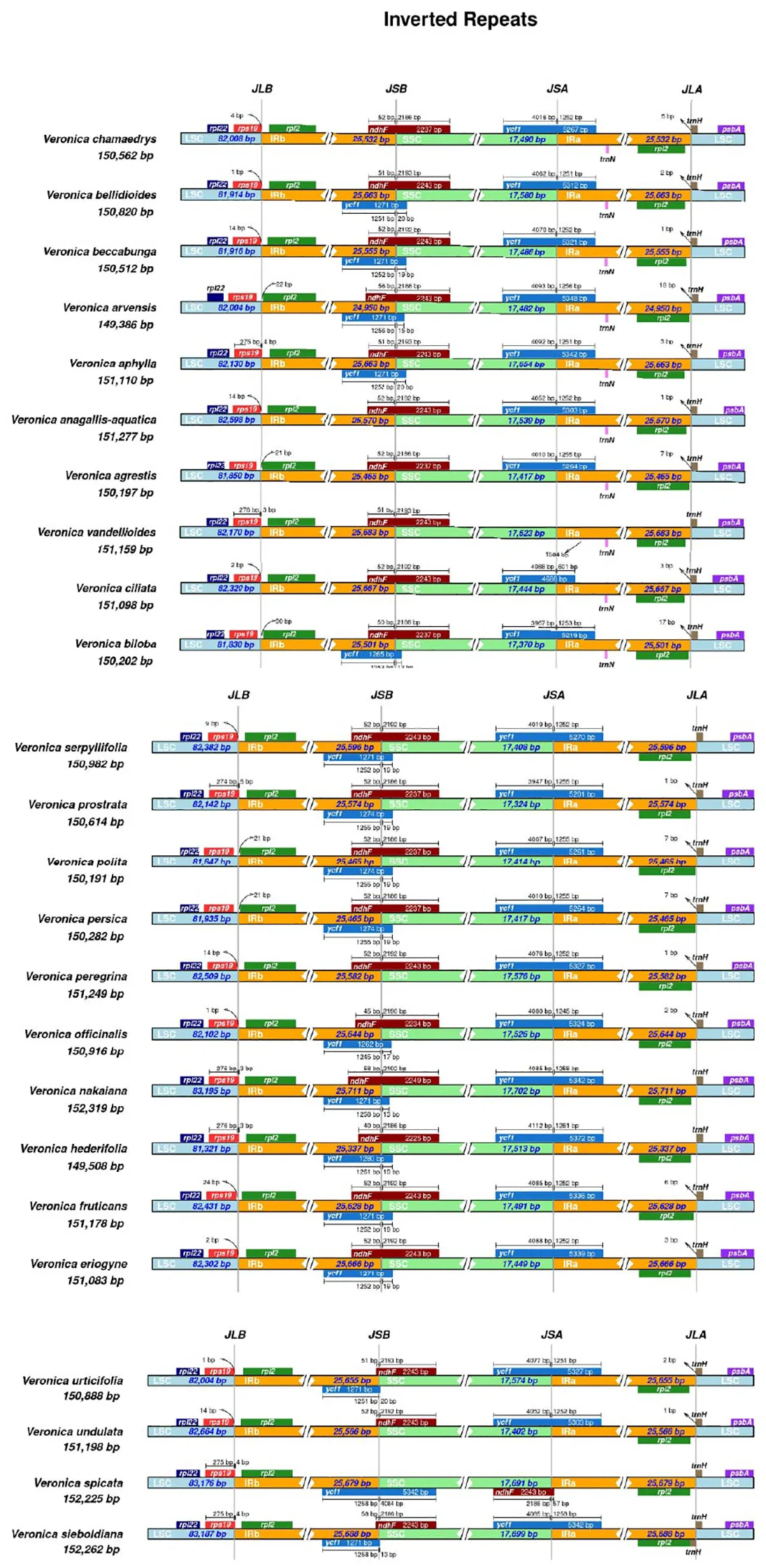
Comparison of inverted repeat/single-copy (IR/SC) border positions across 24 *Veronica* plastomes. Genes are shown as colored boxes, and distances between genes and boundaries are labeled in base pairs (bp).

In general, expansion and contraction of inverted repeat (IR) and single-copy (SC) regions can shift the positions of plastome junctions. Here, IR/SC boundaries were compared among 24 *Veronica* plastomes, comprising the three newly sequenced species and 21 species retrieved from public databases (**Figure 7**). The plastomes showed high conservation in gene order and gene content, but small positional differences were observed at the four IR/SC junctions (JLB, JSB, JSA and JLA). Across the examined species, *rps19* was located at the JLB boundary, and short fragments extended into IRb. The JLA junction was located between the *rpl2* and *trnH.* Except for *Veronica spicata*, *ndhF* spanned the SSC/IRb boundary, with most of the gene located in the SSC region. In most species, *ycf1* was located mainly in the SSC region and partially extended into IRa, and homologous fragments were detected in IRb in several taxa.

### 3.4 Nucleotide diversity and variable hotspots

**Figure 8:**
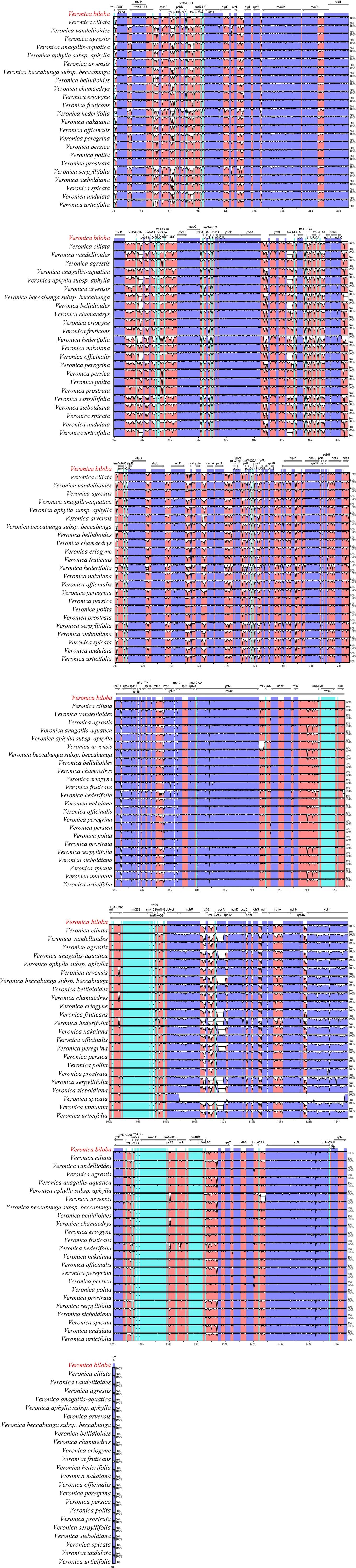
Comparative analysis of the chloroplast genomes of 24 *Veronica* species was conducted. Dark blue bars represent protein-coding genes, light blue bars represent rRNA genes, and red bars represent conserved non-coding sequences. The y-axis scale indicates the percentage of sequence identity (50%–100%). The comparative analysis was performed using the mVISTA software.

mVISTA alignment of 24 *Veronica* plastomes revealed high overall sequence similarity (**Figure 8**). *Veronica* was concentrated in non-coding regions, whereas coding regions were generally more conserved. Several regions with relatively high divergence were detected, including *ycf1*, *ndhF*, *accD* and *rpl32*.

**Figure 9:**
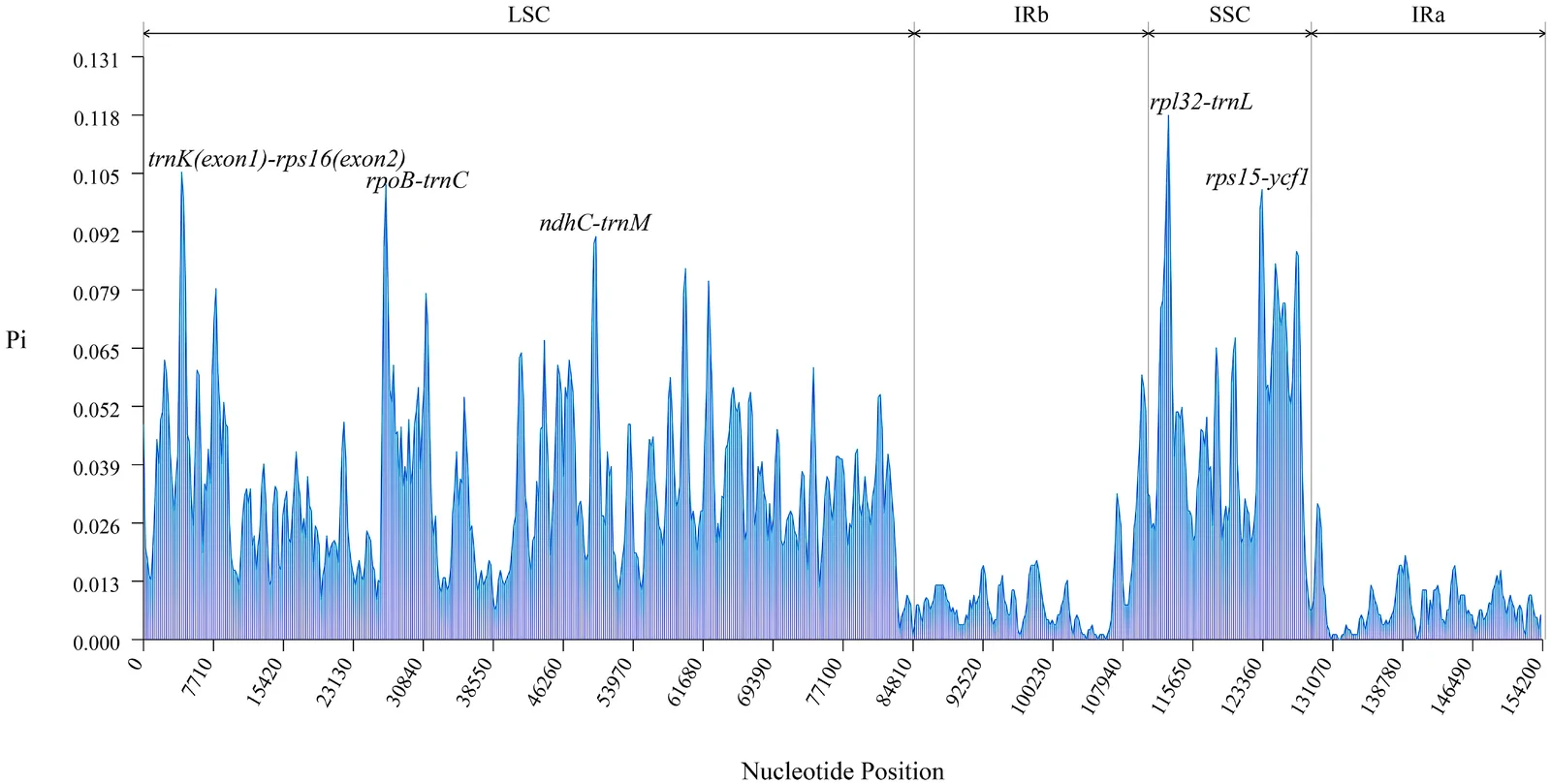
Nucleotide diversity (Pi) values across 24 *Veronica* chloroplast genomes calculated with a 600-bp sliding window and a 200-bp step size.

Sliding-window analysis in the online platform GenePioneer quantified nucleotide diversity across the three *Veronica* plastomes (**Figure 9**). Pi values ranged from 0.00000 to 0.11778, with a genome-wide mean of 0.02736. Genes and intergenic regions with a nucleotide diversity (Pi) greater than three times the genome-wide average (Pi > 0.08208) were identified as mutational hotspots in the chloroplast genome. Under this criterion, a total of 33 windows were identified as hypervariable regions (**Supplementary Table 3**), predominantly located in the large single-copy (LSC) and small single-copy (SSC) regions, whereas the two inverted repeats (IRa and IRb) remained highly conserved. The Pi values of coding regions were found to range from 0.00000 to 0.10278. The maximum value was detected in *rpl32*, and the average Pi value of coding regions was calculated as 0.0274. For non-coding intergenic spacer regions, Pi values were distributed between 0.00000 and 0.11778. The highest nucleotide diversity was observed in the intergenic spacer rpl32-trnL, with an average Pi value of 0.0406. rpl32-trnL (Pi = 0.11778), trnK(exon1)-rps16(exon2) (Pi = 0.10500) and *rpl32* (Pi = 0.10278) were the strongest mutational hotspots. Protein-coding genes including *ycf1*, *ndhF*, *accD*, *matK* and *rpoB* also showed relatively high Pi values, whereas most photosynthesis-related genes, such as *psbA*, *psbD*, *psaA*, *psaB* and *rbcL*, and ribosomal RNA genes such as *rrn16S* and *rrn23S*, were highly conserved.

### 3.5 Codon preference analysis

**Figure 10:**
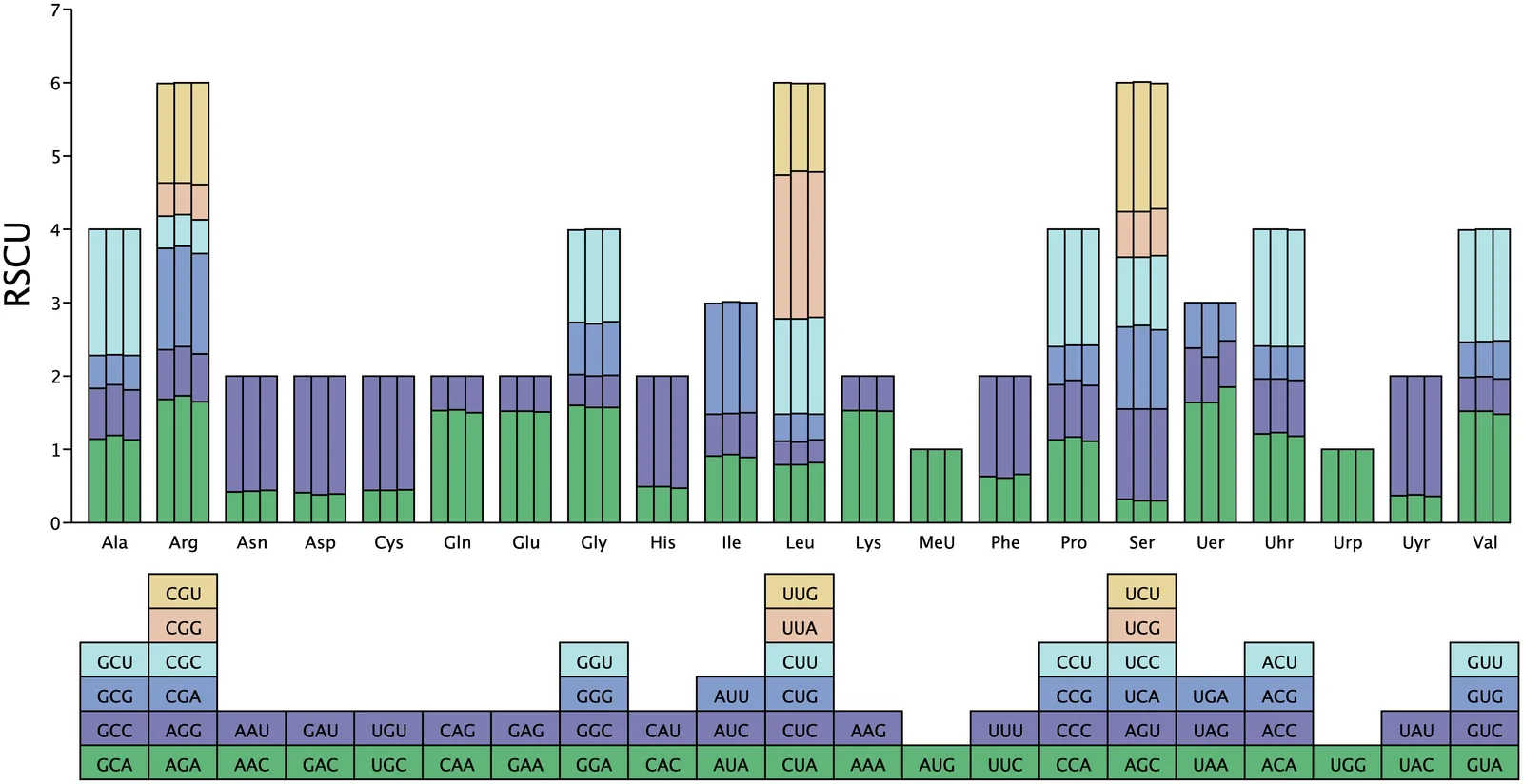
The RSCU values of the 20 amino acids of the complete chloroplast genome of the three *Veronica* taxa and their different codon usages.

Codon preferences were highly similar among the examined species. The analysis revealed that *V. ciliata* and *V. vandellioides* possessed 86 protein-coding genes encoding 64 codons, whereas *V. biloba* harbored 87 protein-coding genes also encoding 64 codons, including three stop codons: UAA, UGA and UAG (**Figure 10**). Leucine was the most abundant amino acid. It was encoded by TTG, TTA, CTT, CTG, CTC and CTA, with codon numbers ranging from 2,081 (in *V. vandellioides*) to 2,266 (in *V. biloba*). In contrast, cysteine was the rarest amino acid. It was encoded by TGT and TGC, with codon counts ranging from 220 (in *V. ciliata*) to 225 (in *V. biloba*).

**Figure 11:**
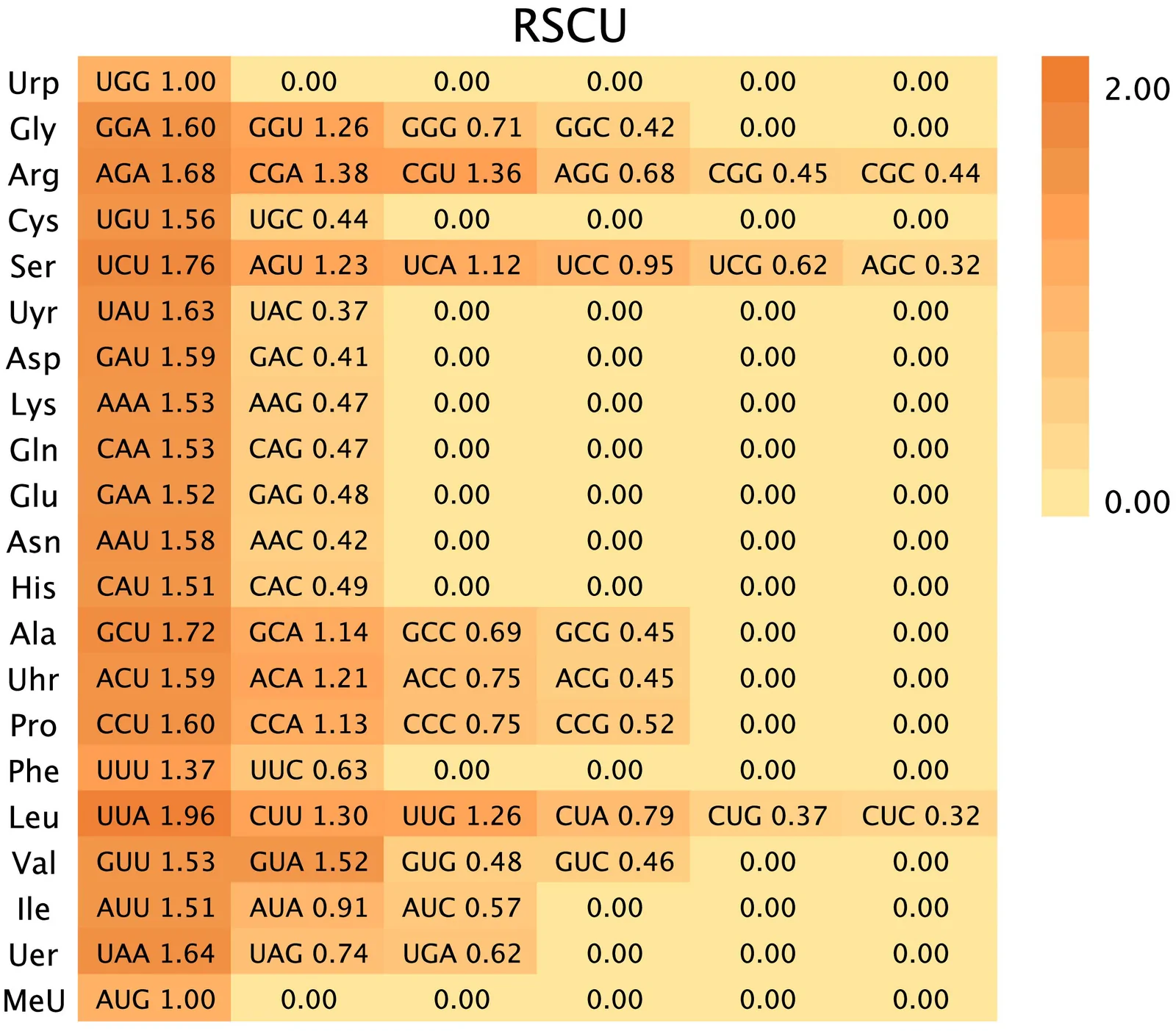
Heatmap of codon usage preferences in the newly sequenced genomes of three *Veronica* chloroplast species.

The codon usage heatmap (**Figure 11**) revealed a strong preference for A/U-ending codons in the *Veronica* chloroplast genomes. Among them, the leucine codon TTA exhibited the highest RSCU value, reaching 1.98. Most preferred codons had RSCU values greater than 1.5, whereas C/G-ending codons were generally used at low frequencies (RSCU < 0.5). The codons for methionine (encoded by ATG) and tryptophan (encoded by TGG) showed no bias, with an RSCU value of 1.00. Among the stop codons, TAA was the most frequently used (RSCU = 1.71), while TAG and TGA were used less frequently.

### 3.6 Phylogenetic analysis

**Figure 12:**
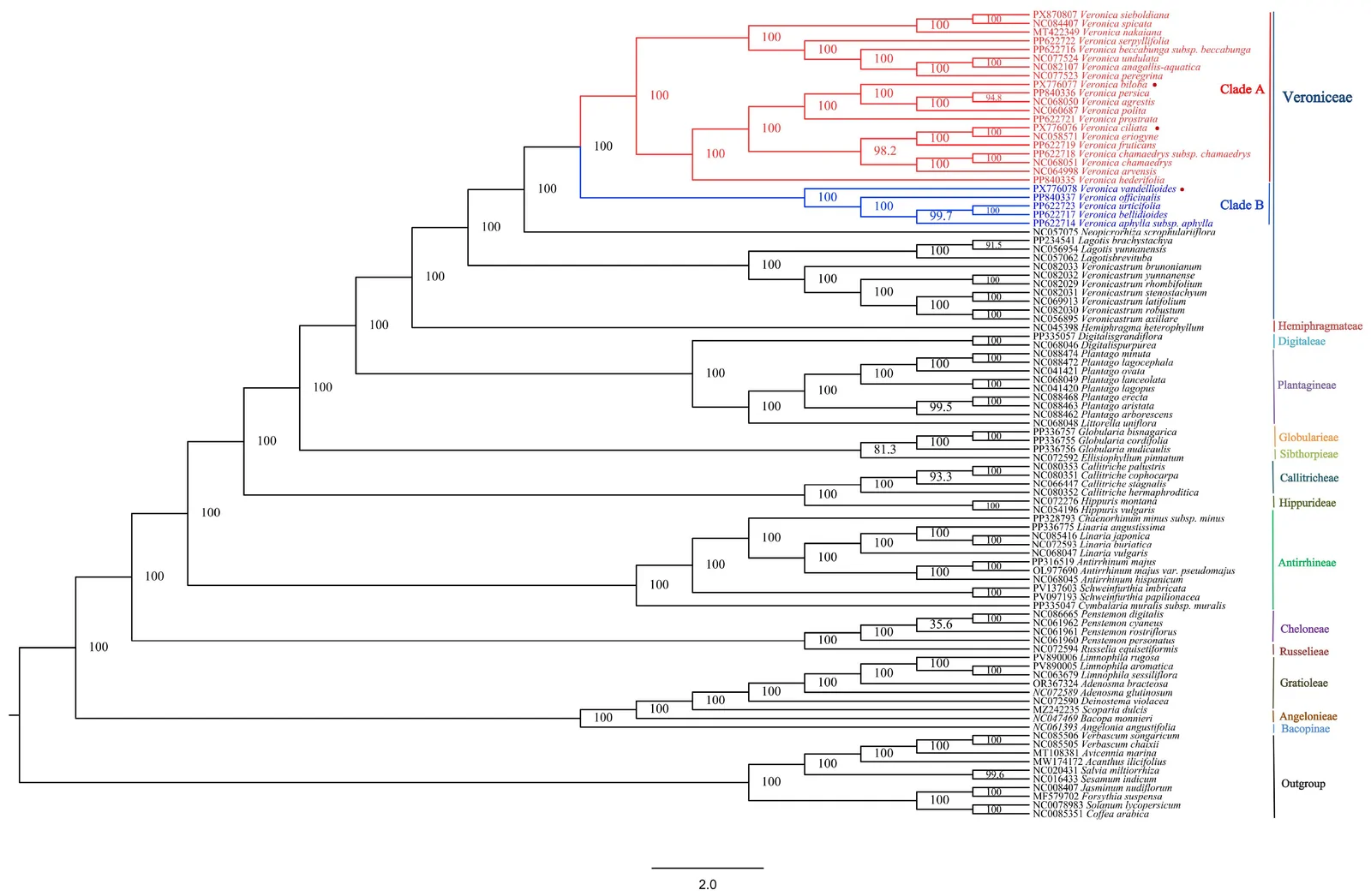
Best maximum likelihood (ML) phylogenetic tree inferred from 93 chloroplast genomes (bootstrap values are labeled on the branches). Species marked with circles are newly sequenced in this study.

In this study, a phylogenetic tree was constructed from 93 complete chloroplast genomes, including 83 Plantaginaceae ingroup taxa and 10 Lamiales outgroups, *Verbascum songaricum*, *Verbascum chaixii, Coffea arabica, Solanum lycopersicum, Jasminum nudiflorum, Avicennia marina, Acanthus ilicifolius, Salvia miltiorrhiza, Sesamum indicum and Forsythia suspensa* (**Figure 12**). The outgroups were placed outside the Plantaginaceae ingroup. Within Plantaginaceae, most major lineages received high bootstrap support, indicating that the complete plastome dataset provided a clear chloroplast phylogenetic framework for the sampled taxa.

Within the Plantaginaceae ingroup, the topology recovered several well-supported tribal or subtribal lineages, including Veroniceae, Hemiphragmateae, Digitaleae, Plantagineae, Globularieae, Sibthorpieae, Callitricheae, Hippurideae, Antirrhineae, Cheloneae, Russelieae, Angelonieae, Bacopinae and Gratioleae. Because this inference is based on chloroplast genomes, the topology should be interpreted as a plastid genealogy. It may not fully capture species histories in groups affected by hybridization, introgression or incomplete lineage sorting. The sampled members of Veroniceae formed a well-supported plastid clade that included *Veronica*, *Veronicastrum*, *Neopicrorhiza* and *Lagotis*. In the recovered topology, *Veronica* was strongly supported as monophyletic, and its relationships with closely related genera were described according to the topology shown in **Figure 12** rather than as a general resolution of all intergeneric relationships in the tribe.

The sampled *Veronica* species formed a strongly supported monophyletic group in the chloroplast tree. Within *Veronica*, two major plastid clades were recovered. In Clade A, *V. ciliata* clustered with *Veronica eriogyne* with strong support, while *Veronica persica*, *Veronica agrestis* and *Veronica polita* formed a stable subclade, with *V. biloba* recovered as sister to that subclade. In Clade B, *Veronica urticifolia*, *Veronica bellidioides*, *Veronica officinalis* and *Veronica aphylla* subsp. *aphylla* formed a highly supported subclade, and *V. vandellioides* was placed within this clade.

Therefore, the plastid phylogenetic positions of the three newly sequenced species were clarified within *Veronica* based on the well-resolved tree topology. This topology was further utilized to perform ancestral state reconstruction of diagnostic floral morphological traits. These results provide chloroplast genomic evidence and morphological evidence for their placement, but they should not be interpreted as resolving all taxonomic questions within the genus without additional nuclear data.

### 3.7 Morphological trait analysis

**Figure 13:**
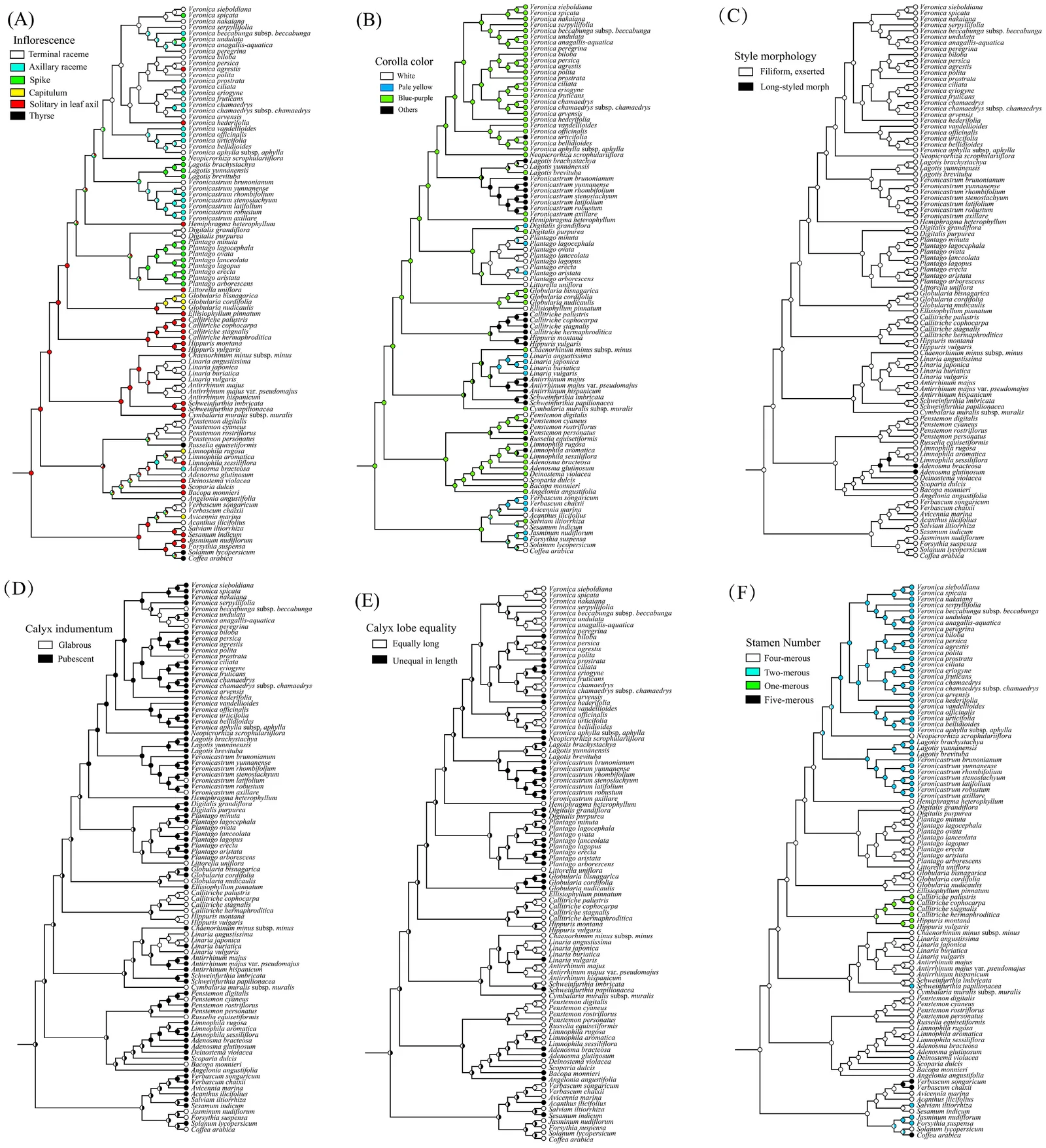
Ancestral state reconstruction of six key morphological characters using the method of likelihood ancestral states with the Mk1 model. **(A)** inflorescence type; **(B)** corolla color; **(C)** style morphology; **(D)** calyx indumentum; **(E)** sepal equality; **(F)** stamen number.

Ancestral character reconstruction was performed on six key morphological traits of Plantaginaceae in this study (**Figure 13**). The investigated characters included inflorescence (A), corolla color (B), style morphology (C), calyx indumentum (D), calyx lobe equality (E) and stamen number (F). Terminal raceme were supported as the ancestral inflorescence type of Plantaginaceae by the reconstruction results. The early formation of this fundamental inflorescence architecture during the initial divergence of the family was therefore demonstrated. Lateral racemes, spikes, capitula, solitary axillary flowers and thyrses evolved independently across multiple lineages within the family. Blue-purple corolla was inferred as the ancestral state for *Veronica*. In contrast, white, pale yellow and other corolla color phenotypes originated repeatedly in separate clades. The long-styled morph was resolved as the ancestral style morphology of Plantaginaceae, and high statistical support was obtained at basal phylogenetic nodes. Filiform exserted styles evolved convergently in numerous derived lineages. Glabrous calyx was supported as the ancestral state of calyx indumentum. An 85% posterior probability was obtained at the basal node of Plantaginaceae. Equal calyx lobes were inferred as the ancestral condition for the family. Unequal calyx lobes arose independently evolution in multiple genera. This feature was recognized as a vital diagnostic character for species delimitation in groups such as *Veronica*. The four-stamen condition was strongly supported as the ancestral stamen number within *Veronica*. This trait was consistent with the core morphological synapomorphies of Plantaginaceae. Reductions to two or one stamens were observed to evolve repeatedly in numerous derived clades.

This analysis provided critical insights into the morphological evolution and diversification history of Plantaginaceae (especially *Veronica*). These valuable findings were derived from the combined plastid phylogenetic framework and ancestral character reconstruction results described above. Reliable evolutionary context for interpreting morphological variation of the three newly sequenced *Veronica* species was offered by the reconstructed ancestral states. The diagnostic value of these morphological traits for taxonomic delimitation within the genus was further clarified simultaneously.

### 3.8 Divergence time estimation

**Figure 14:**
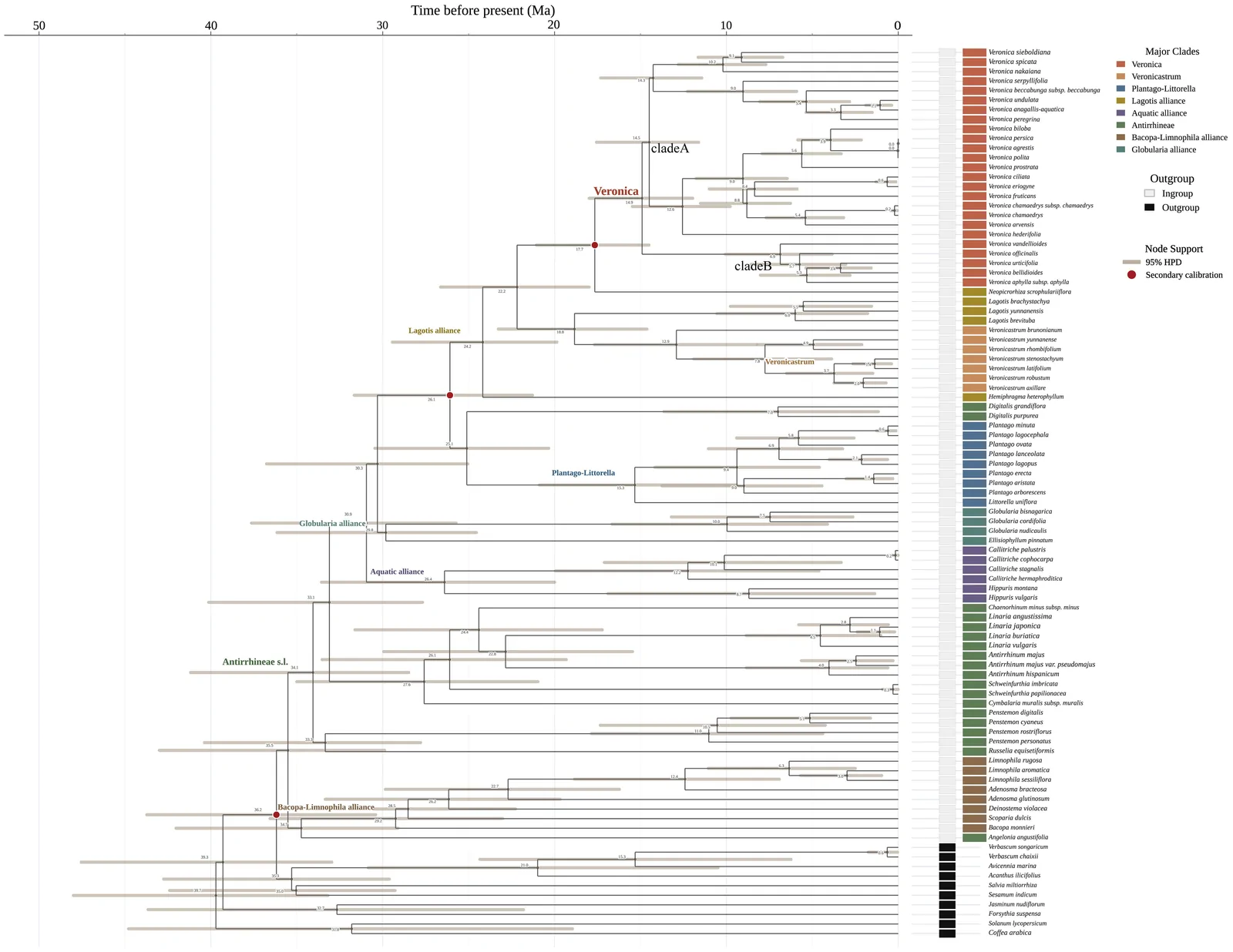
Divergence time estimation of the genus *Veronica* based on the complete chloroplast genome dataset. The BEAST analysis was performed using the complete chloroplast genome data. The yellow bars represent the 95% highest posterior density (HPD) intervals, and the red circles indicate the secondary calibration points.

Based on the calibrated chloroplast phylogeny, divergence times of lineages within *Veronica* were estimated (**Figure 14**). Because the crown node of *Veronica* was constrained at 14.9 Ma, this age should be interpreted as calibration-dependent rather than as an independent estimate. Under this calibrated framework, Clade A and Clade B were estimated at approximately 14.5 Ma and 6.9 Ma, respectively. The estimated divergence times of *V. biloba*, *V. ciliata* and *V. vandellioides* were approximately 3.9 Ma, 0.6 Ma and 6.9 Ma, respectively.

## 4 Discussion

### 4.1 Plastome structure and characteristics analysis

The three newly sequenced *Veronica* plastomes were highly similar in structure, gene order, genome size and gene count, which is consistent with previous reports for *Veronica* and other Plantaginaceae plastomes (Choi et al., 2016; Maurya et al., 2020; H Liu et al., 2022; Jia et al., 2023; Zhao et al., 2023; Zhang et al., 2025). Their genome sizes differed by less than 1 kb, and GC content varied only from 37.9% to 38.2%. These small differences suggest that plastome evolution in the sampled *Veronica* species has been dominated by structural conservation rather than large rearrangements. The value of the newly generated plastomes therefore lies less in detecting novel plastome architecture and more in improving species-level sampling for comparative and phylogenetic analyses within the genus (Liu et al., 2022; Guo et al., 2025; Olivar et al., 2025).

### 4.2 Repeat sequence analysis

The SSR results show that *Veronica* plastomes share an AT-rich repeat profile, but they differ in SSR abundance and motif composition among species. The range of 31–61 SSR loci should not be described as numerically similar; rather, it indicates moderate variation in repeat number across the sampled plastomes. The dominance of A/T mononucleotide repeats is consistent with the AT-rich nature of plastid genomes (Hai et al., 2024), whereas species-specific motifs, such as the pentanucleotide repeat detected in *V. biloba* and the C/G motif detected in *V. vandellioides*, may be useful for developing chloroplast markers after validation in population-level samples. These repeat features provide practical targets for future marker screening (López-González et al., 2015), although primer design and polymorphism testing are still required before they can be used for species identification or population genetics.

### 4.3 Expansion and contraction of the border regions

The IR/SC boundary comparison indicates that *Veronica* plastomes did not undergo large-scale IR expansion or contraction, but small shifts around boundary-associated genes were detected. Such localized variation is common in angiosperm plastomes and can contribute to minor length differences among closely related species (Kim and Lee, 2004; Song et al., 2017). In this study, genes near the SSC/IR boundaries, especially *ndhF*, *ycf1* and *rps19*, showed the most informative boundary changes. These findings refine the interpretation of plastome conservation in *Veronica*: the overall architecture is stable, but boundary regions still retain enough variation to be useful in comparative plastomics.

### 4.4 Sequence divergence and hotspot

The hotspot analysis provides a more quantitative basis for selecting candidate markers in *Veronica*. Using Pi > 0.08208 as the threshold, rpl32-trnL, trnK(exon1)-rps16(exon2) and *rpl32* were identified as the strongest hotspots, while *ycf1*, *ndhF*, *accD*, *matK* and *rpoB* also showed elevated variation. These regions include both intergenic spacers and protein-coding genes, making them suitable candidates for species-level barcoding or population-level marker development (Hai et al., 2024). However, their practical value should be verified by primer design, amplification success and intraspecific sampling. In contrast, photosynthesis-related and ribosomal RNA genes showed low sequence divergence, suggesting strong functional constraint on core plastid genes.

### 4.5 Codon usage analysis

Consistent codon usage patterns were observed across the three species (**Figures 10** and **11**). Leucine was identified as the most abundant amino acid, whereas cysteine was the least abundant. Additionally, most codons ending with A/U exhibited RSCU values greater than 1, indicating a strong preference for A/U-ending codons. This preference is consistent with the high AT content of chloroplast genomes and the requirements for efficient expression of photosynthesis-related genes, as reported in previous studies (Morton, 1999; Choi et al., 2016; Jia et al., 2022; Wu et al., 2023; Hai et al., 2024). Codon usage bias was highly similar among different species.

### 4.6 Plastid phylogeny and ancestral floral trait evolution of *Veronica*

The chloroplast phylogeny recovered *Veronica* as a well-supported monophyletic lineage and clarified the plastid placement of the three newly sequenced species. This improves taxon sampling within the genus and provides a more stable plastome framework for comparing newly generated and published chloroplast genomes. These findings are consistent with several previous plastid and multilocus studies that support the distinctiveness of major *Veronica* lineages (Albach et al., 2005a; Albach and Meudt, 2010; Padilla-García et al., 2018; Hai et al., 2024). Within Veroniceae, the sampled *Veronica* plastomes formed two major clades, and the three newly sequenced species were placed in different positions within this framework. *V. ciliata* was closely associated with *V. eriogyne*, *V. biloba* was placed near the *V. persica*-*V. agrestis*-*V. polita subclade*, and *V. vandellioides* was recovered within Clade B. This result is congruent with traditional taxonomic classifications based on floral morphology and phylogenetic inferences obtained from Sanger-sequenced short DNA fragments (Albach et al., 2005a).

Ancestral state reconstruction was performed for six core diagnostic floral traits based on the plastid phylogenetic tree generated in this study. Terminal raceme, blue-purple corolla, long-styled morph, glabrous calyx, equal calyx lobes and four stamens were inferred as the ancestral character states of Plantaginaceae and the genus *Veronica*. The widespread retention of these plesiomorphic traits across phylogenetically distant lineages within the genus suggests that they constitute a conserved floral ground plan maintained since the early divergence of *Veronica*. Such conservation is likely driven by functional constraints associated with generalized pollination systems and fundamental reproductive assurance (Albach et al., 2004b). In particular, blue-purple corolla pigmentation represents a common characteristic among bee-pollinated Lamiales. This trait may have been conserved owing to its capacity to attract a broad spectrum of generalist pollinators, which reduces selective pressure toward derived color shifts in lineages occupying stable habitats. High congruence was detected between our character reconstructions and the outcomes published by Albach et al. (2004b). The recurrent occurrence of these traits in distantly related taxa cannot be attributed to shared ancestry. Instead, this pattern is consistent with convergent adaptation to similar ecological conditions, including the evolution of selfing syndromes in annual ruderal lineages (Muller and Albach, 2010) and pollinator specialization in alpine populations (Thomas et al., 2023).

Broad congruence was obtained between the present reconstructions and findings from earlier comparative morphological investigations of Plantaginaceae, as well as previous analyses focusing on trait evolution within Veroniceae. A sister relationship between *V. biloba* and the subclade consisting of *V. persica*, *V. agrestis* and *V. polita* was recovered in our topology. This topological pattern was matched by the results reported by Albach et al. (2004a), who recognized these annual species as a distinct paraphyletic group with recurrent evolutionary trends and molecular convergence. *V. vandellioides* was placed within Clade B in our plastid tree. This placement agreed with the research of Scatigna et al. (2022) on Gratioleae, in which most characters applied to circumscribe broad lineages were identified as plesiomorphic, while combined floral traits were proven reliable diagnostic markers for generic delimitation. Consistency between our chloroplast-derived phylogeny and traditional taxonomic schemes based on floral morphology was also documented in the present work. This observation echoed the viewpoints raised by Albach et al. (2004b), who stated that morphological features such as inflorescence type, calyx lobe number and capsule shape were evolutionarily labile, yet valuable taxonomic information could still be supplied when these traits were interpreted within a molecular phylogenetic framework.

A distinct association between divergence chronology and morphological differentiation is revealed by integrating ancestral state reconstructions and divergence time estimates. The earlier-diverging Clade A retains the full ancestral suite, whereas the more recently diversified Clade B exhibits greater morphological disparity. This observation suggests a systematic difference in the tempo of floral evolution between the two major *Veronica* lineages.

The plastid phylogenetic placements of three newly sequenced *Veronica* species were precisely clarified in this study. Combined plastid genomic and ancestral morphological reconstruction data were adopted to support their taxonomic status. However, the tree is based on maternally inherited plastomes and therefore represents the chloroplast history of the sampled taxa. Nuclear data and broader population sampling are still needed to test relationships that may have been affected by hybridization, introgression or incomplete lineage sorting.

### 4.7 Divergence time estimation analysis

The divergence-time analysis should be interpreted in light of its calibration design. Because the crown node of *Veronica* was constrained at 14.9 Ma following previous studies, the estimated age of this node mainly reflects the calibration setting rather than a fully independent result. Within this calibrated framework, the timing of subsequent splits provides a useful relative timescale for comparing the three newly sequenced species and their relatives. The inferred Miocene to Pleistocene divergences are broadly compatible with previous studies that linked *Veronica* diversification to climatic cooling, habitat shifts and mountain-associated diversification in Eurasia (Jiménez-Moreno et al., 2010; Wang et al., 2016; Jia and Bartish, 2018).

The two major clades differ not only in morphological conservatism but also in their estimated divergence ages. Clade A, estimated to have diverged approximately 14.5 Ma, has a longer independent evolutionary history, and this longer history likely facilitated the retention of ancestral morphological characters. By contrast, Clade B, estimated to have diverged approximately 6.9 Ma, diversified more recently, and this more recent diversification coincided with the late Miocene expansion of open habitats and seasonal climates (Jiménez-Moreno et al., 2010). These conditions may have favored rapid morphological divergence and the evolution of novel trait combinations. The observed temporal asymmetry parallels patterns documented in other plant radiations, with the older clade exhibiting more conserved morphology and the younger clade showing elevated disparity. This asymmetry may reflect differences in the ecological opportunities available to each lineage at the time of its origin (Meudt et al., 2015). This pattern is largely consistent with the phylogenetic structure of *Veronica* observed in previous studies (Albach and Chase, 2004; Albach et al., 2004a, c; Bardy et al., 2010; Choi et al., 2016).

Among the newly sequenced species, *V. biloba* was estimated to have diverged at approximately 3.9 Ma. This period witnessed late Neogene climatic fluctuations and increasing aridification in parts of Central Asia, as well as the expansion of seasonally dry habitats during the Pliocene (Jiménez-Moreno et al., 2010; Wang et al., 2016). These environmental changes may have favored the evolution of annual or short-lived life-history strategies, potentially driving convergent shifts in floral morphology. Nevertheless, because the present analysis is based on plastid data alone, these evolutionary interpretations require further validation with nuclear markers to rule out chloroplast capture or incomplete lineage sorting (Khan et al., 2024).

The extremely recent origin of *V. ciliata* was dated to the Late Pleistocene. This implies that its speciation and subsequent population expansion were likely triggered by climatic fluctuations associated with Quaternary glacial cycles. This interpretation is supported by multiple lines of evidence from other *Veronica* studies. First, divergence times within 1–2 Ma were estimated for *Veronica* subg. *Pseudolysimachium* in the Altai region by Khan et al. (2024), and high levels of historical gene flow were detected. Second, mountain uplift was demonstrated to be a major driver of diversification in New Zealand *Veronica* sect. *Hebe* by Thomas et al. (2023), with *in situ* diversification rates in montane habitats approximately eight times higher than in lowland habitats around 2–3 Ma. Third, the *Veronica chamaedrys* complex in southeastern Europe was found to have diversified during the Middle Pleistocene by Bardy et al. (2010), with ongoing divergence over the last 400,000 years. Collectively, these studies suggest that Pleistocene climatic oscillations were a general driver of rapid speciation in *Veronica*. This pattern is particularly consistent with the recent origin of *V. ciliata* in the high-elevation mountains of Southwest China, where glaciation-caused alpine habitat fragmentation in alpine regions may have played a key role.

In contrast, the divergence time of *V. vandellioides* is substantially older (6.9 Ma). This species occupies a relatively isolated position in the phylogenetic tree (**Figure 12**), which corresponds to its distinctive morphological characters, suggesting that it may represent an early-diverging and independent lineage within the genus *Veronica* (Albach et al., 2004b, 2008; Doostmohammadi et al., 2022). Its ancient divergence coupled with morphological uniqueness implies that *V. vandellioides* survived as a relictual lineage in stable montane refugia since the late Miocene. It was sheltered from climatic oscillations that accelerated diversification of other congeneric lineages.

Taken together, the divergence-time estimates provide a calibrated temporal framework for interpreting plastid diversification in *Veronica*. They suggest that the three newly sequenced species represent different temporal depths within the genus, ranging from a recent split in *V. ciliata* to older divergence in *V. vandellioides*. At the same time, the dependence on secondary calibrations, the use of chloroplast genomes only, and the absence of nuclear data mean that these estimates should be treated as a working timescale rather than a complete reconstruction of species history. Future analyses integrating nuclear loci, broader population sampling and explicit biogeographic models will be needed to test the evolutionary scenarios suggested by the plastid tree.

## 5 Conclusions

In this study, the complete chloroplast genomes of three *Veronica* species were sequenced and compared with published plastomes. The three genomes were highly conserved in size, structure, gene content and overall organization, with total lengths of 150,202 bp for *V. biloba*, 151,098 bp for *V. ciliata* and 151,159 bp for *V. vandellioides*. They contained 130–132 unique genes, including 86–87 protein-coding genes, 36–37 tRNA genes and 8 rRNA genes. SSR and repeat analyses showed both conserved AT-rich repeat patterns and species-level differences in motif composition. IR/SC boundary comparisons revealed no large-scale IR expansion or contraction, but localized shifts near boundary-associated genes such as *ndhF*, *ycf1* and *rps19*. Nucleotide diversity analysis identified rpl32-trnL, trnK(exon1)-rps16(exon2), *rpl32*, *ycf1*, *ndhF*, *accD*, *matK* and *rpoB* as candidate highly variable regions. The ML tree based on 83 Plantaginaceae ingroup taxa and 10 Lamiales outgroups recovered *Veronica* as a well-supported plastid clade and clarified the chloroplast positions of *V. biloba*, *V. ciliata* and *V. vandellioides*. Ancestral character states of *Veronica* were inferred through reconstruction analyses. Blue-purple corollas and four stamens were resolved as the ancestral floral features of this genus. Terminal racemes, filiform exserted styles, glabrous calyces and equal calyx lobes, by contrast, were recognized as plesiomorphic traits shared by all members of Plantaginaceae. Divergence-time estimation, interpreted under the specified calibration scheme, placed these three species at different temporal depths within *Veronica*. *V. biloba* diverged around 3.9 Ma, while *V. ciliata* and *V. vandellioides* diverged around 0.6 Ma and 6.9 Ma, respectively.

This study provides newly assembled plastome resources and a revised chloroplast phylogenetic framework for *Veronica*. The results support the use of complete plastomes for species-level comparison and marker discovery in the genus. However, our phylogeny and dating results were derived from chloroplast genomes only. Despite the integration of morphological analyses and ancestral character reconstruction in this work, nuclear markers and denser taxon sampling remain necessary to support subsequent taxonomic revisions and evolutionary history.

## Data Availability

The datasets presented in this study can be found in online repositories. The names of the repository/repositories and accession number(s) can be found below: https://www.ncbi.nlm.nih.gov/, PX776076.1 https://www.ncbi.nlm.nih.gov/, PX776077.1 https://www.ncbi.nlm.nih.gov/, PX776078.1.
