# Supplementary material for: Insights into phylogenetic relationships of Veronica species (Plantaginaceae) based on comparative chloroplast genomics
Source: Front Plant Sci. 2026 Aug 18;17:1885890. doi: 10.3389/fpls.2026.1885890 (PMC13529719; doi:10.3389/fpls.2026.1885890)
Supplement: Supplementary file 1 [file DataSheet2.docx]

Supplementary Material

# 1 Supplementary Figures and Tables

## 1.1 Supplementary Figures

**Supplementary Figure 1.** Sequencing depth and coverage map of the *Veronica vandellioides* chloroplast genome. The blue line represents the mean depth calculated across 500 bp sliding windows. The total genome length is 151,159 bp, with an average depth of 2,202.09×, a maximum depth of 3,275×, and a minimum depth of 781×. The relatively stable coverage pattern demonstrates consistent sequencing quality across all genomic regions.

**Supplementary Figure 2.** Sequencing depth and coverage map of the *Veronica biloba* chloroplast genome. The blue line represents the mean depth calculated across 500 bp sliding windows. The total genome length is 150,202 bp, with an average depth of 3,171.76×, a maximum depth of 4,355×, and a minimum depth of 1,017×. The relatively stable coverage pattern demonstrates consistent sequencing quality across all genomic regions.

**Supplementary Figure 3.** Sequencing depth and coverage map of the *Veronica ciliata* chloroplast genome. The blue line represents the mean depth calculated across 500 bp sliding windows. The total genome length is 151,098 bp, with an average depth of 1,545.53×, a maximum depth of 2,075×, and a minimum depth of 621×. The relatively stable coverage pattern demonstrates consistent sequencing.

## 1.2 Supplementary Table

**Supplementary Table 1.** Sequencing quality statistics of chloroplast genomes for three *Veronica* species

| Species Name | Total Raw Sequencing Data (Gbp) | Q20 Base Percentage (%) | Q30 Base Percentage (%) | Number of Mapped Reads (M) | Mapping Rate (%) |
| --- | --- | --- | --- | --- | --- |
| *Veronica vandellioides* | 10.64 | 98.71 | 95.32 | 34.5 | 98.8 |
| *Veronica biloba* | 6.21 | 98.10 | 92.03 | 20.12 | 98.61 |
| *Veronica ciliata* | 7.02 | 98.10 | 91.41 | 22.85 | 98.55 |

**Supplementary Table 2.** Morphological matrix of 93 species.

| GenBank | Species | Inflorescence 0 Terminal raceme 1 Axillary raceme 2 Spike 3 Capitulum 4 Solitary in leaf axil 5 Thyrse | Corolla color 0 White 1 Pale yellow 2 Blue-purple 3 Others | Style morphology 0 Filiform, exserted 1 Long-styled morph | Calyx indumentum 0 Glabrous 1 Pubescent | Calyx lobe equality 0 Equally long 1 Unequal in length | Stamen Number 0 Four-merous 1 Two-merous 2 One-merous 3 Five-merous |
| --- | --- | --- | --- | --- | --- | --- | --- |
| OR367324.1 | *Adenosma bracteosa* | 1 | 2 | 1 | 1 | 1 | 0 |
| NC_072589.1 | *Adenosma glutinosum* | 0 | 2 | 1 | 1 | 1 | 0 |
| NC_061393.1 | *Angelonia angustifolia* | 0 | 2 | 0 | 1 | 1 | 0 |
| NC_068045.1 | *Antirrhinum hispanicum* | 0 | 3 | 0 | 1 | 0 | 0 |
| OL977690.1 | *Antirrhinum majus var. pseudomajus* | 0 | 3 | 0 | 1 | 0 | 0 |
| PP316519.1 | *Antirrhinum majus* | 0 | 3 | 0 | 1 | 0 | 0 |
| NC_047469.1 | *Bacopa monnieri* | 4 | 2 | 0 | 0 | 1 | 0 |
| NC_080351.1 | *Callitriche cophocarpa* | 4 | 3 | 0 | 0 | 0 | 2 |
| NC_080352.1 | *Callitriche hermaphroditica* | 4 | 3 | 0 | 0 | 0 | 2 |
| NC_080353.1 | *Callitriche palustris* | 4 | 3 | 0 | 0 | 0 | 2 |
| NC_066447.1 | *Callitriche stagnalis* | 4 | 3 | 0 | 0 | 0 | 2 |
| PP328793.1 | *Chaenorhinum minus subsp. minus* | 4 | 2 | 0 | 1 | 0 | 0 |
| PP335047.1 | *Cymbalaria muralis subsp. muralis* | 4 | 2 | 0 | 0 | 0 | 0 |
| NC_072590.1 | *Deinostema violacea* | 4 | 2 | 0 | 1 | 0 | 1 |
| PP335057.1 | *Digitalis grandiflora* | 0 | 1 | 0 | 1 | 1 | 0 |
| NC_068046.1 | *Digitalis purpurea* | 0 | 2 | 0 | 1 | 1 | 0 |
| NC_072592.1 | *Ellisiophyllum pinnatum* | 4 | 0 | 0 | 1 | 0 | 0 |
| PP336757.1 | *Globularia bisnagarica* | 3 | 2 | 0 | 1 | 1 | 0 |
| PP336755.1 | *Globularia cordifolia* | 3 | 2 | 0 | 1 | 1 | 0 |
| PP336756.1 | *Globularia nudicaulis* | 3 | 2 | 0 | 0 | 1 | 0 |
| NC_045398.1 | *Hemiphragma heterophyllum* | 4 | 2 | 0 | 1 | 0 | 0 |
| NC_072276.1 | *Hippuris montana* | 4 | 3 | 0 | 0 | 0 | 2 |
| NC_054196.1 | *Hippuris vulgaris* | 4 | 3 | 0 | 0 | 0 | 2 |
| PP234541.1 | *Lagotis brachystachya* | 2 | 3 | 0 | 1 | 1 | 1 |
| NC_057062.1 | *Lagotis brevituba* | 2 | 2 | 0 | 1 | 0 | 1 |
| NC_056954.1 | *Lagotis yunnanensis* | 2 | 0 | 0 | 1 | 0 | 1 |
| PV890005.1 | *Limnophila aromatica* | 0 | 3 | 0 | 1 | 0 | 0 |
| PV890006.1 | *Limnophila rugosa* | 3 | 2 | 0 | 1 | 0 | 0 |
| NC_063679.1 | *Limnophila sessiliflora* | 4 | 2 | 0 | 1 | 0 | 0 |
| PP336775.1 | *Linaria angustissima* | 0 | 1 | 0 | 0 | 0 | 0 |
| NC_072593.1 | *Linaria buriatica* | 0 | 1 | 0 | 1 | 0 | 0 |
| NC_085416.1 | *Linaria japonica* | 0 | 1 | 0 | 0 | 0 | 0 |
| NC_068047.1 | *Linaria vulgaris* | 0 | 1 | 0 | 0 | 1 | 0 |
| NC_068048.1 | *Littorella uniflora* | 4 | 0 | 0 | 0 | 0 | 0 |
| NC_057075.1 | *Neopicrorhiza scrophulariiflora* | 2 | 2 | 0 | 1 | 1 | 0 |
| NC_061962.1 | *Penstemon cyaneus* | 0 | 2 | 0 | 0 | 0 | 0 |
| NC_086665.1 | *Penstemon digitalis* | 0 | 0 | 0 | 1 | 0 | 0 |
| NC_061960.1 | *Penstemon personatus* | 0 | 2 | 0 | 1 | 0 | 0 |
| NC_061961.1 | *Penstemon rostriflorus* | 0 | 3 | 0 | 1 | 0 | 0 |
| NC_088462.1 | *Plantago arborescens* | 2 | 0 | 0 | 1 | 1 | 0 |
| NC_088463.1 | *Plantago aristata* | 2 | 1 | 0 | 1 | 1 | 0 |
| NC_088468.1 | *Plantago erecta* | 2 | 0 | 0 | 1 | 1 | 0 |
| NC_088472.1 | *Plantago lagocephala* | 2 | 1 | 0 | 1 | 1 | 0 |
| NC_041420.1 | *Plantago lagopus* | 2 | 0 | 0 | 1 | 1 | 0 |
| NC_068049.1 | *Plantago lanceolata* | 2 | 0 | 0 | 1 | 1 | 0 |
| NC_088474.1 | *Plantago minuta* | 2 | 0 | 0 | 1 | 0 | 0 |
| NC_041421.1 | *Plantago ovata* | 2 | 0 | 0 | 0 | 0 | 0 |
| NC_072594.1 | *Russelia equisetiformis* | 5 | 3 | 0 | 0 | 0 | 0 |
| PV137603.1 | *Schweinfurthia imbricata* | 4 | 3 | 0 | 1 | 0 | 0 |
| PV097193.1 | *Schweinfurthia papilionacea* | 4 | 3 | 0 | 1 | 1 | 1 |
| MZ242235.1 | *Scoparia dulcis* | 4 | 0 | 0 | 1 | 0 | 0 |
| NC_068050.1 | *Veronica agrestis* | 4 | 2 | 0 | 1 | 1 | 1 |
| NC_082107.1 | *Veronica anagallis-aquatica* | 1 | 2 | 0 | 0 | 0 | 1 |
| PP622714.1 | *Veronica aphylla subsp. aphylla* | 0 | 2 | 0 | 1 | 1 | 1 |
| NC_064998.1 | *Veronica arvensis* | 0 | 2 | 0 | 1 | 1 | 1 |
| PP622716.1 | *Veronica beccabunga* | 1 | 2 | 0 | 0 | 0 | 1 |
| PP622717.1 | *Veronica bellidioides* | 0 | 2 | 0 | 1 | 0 | 1 |
| PX776077.1 | *Veronica biloba* | 0 | 2 | 0 | 1 | 1 | 1 |
| NC_068051.1 | *Veronica chamaedrys* | 1 | 2 | 0 | 1 | 0 | 1 |
| PP622718.1 | *Veronica chamaedrys subsp. chamaedry* | 1 | 2 | 0 | 1 | 0 | 1 |
| PX776076.1 | *Veronica ciliata* | 0 | 2 | 0 | 1 | 1 | 1 |
| NC_058571.1 | *Veronica eriogyne* | 1 | 2 | 0 | 1 | 1 | 1 |
| PP622719.1 | *Veronica fruticans* | 0 | 2 | 0 | 1 | 0 | 1 |
| PP840335.1 | *Veronica hederifolia* | 4 | 2 | 0 | 1 | 1 | 1 |
| MT422349.1 | *Veronica nakaiana* | 0 | 2 | 0 | 1 | 0 | 1 |
| NC_077523.1 | *Veronica peregrina* | 0 | 2 | 0 | 0 | 0 | 1 |
| PP840336.1 | *Veronica persica* | 0 | 2 | 0 | 1 | 0 | 1 |
| NC_060687.1 | *Veronica polita* | 0 | 2 | 0 | 1 | 0 | 1 |
| PP622721.1 | *Veronica prostrata* | 1 | 2 | 0 | 0 | 1 | 1 |
| PP622722.1 | *Veronica serpyllifolia* | 0 | 2 | 0 | 1 | 0 | 1 |
| PX870807.1 | *Veronica sieboldiana* | 0 | 2 | 0 | 1 | 0 | 1 |
| NC_084407.1 | *Veronica spicata*（Pseudolysimachion spicatum） | 2 | 2 | 0 | 1 | 0 | 1 |
| NC_077524.1 | *Veronica undulata* | 2 | 2 | 0 | 1 | 0 | 1 |
| PP622723.1 | *Veronica urticifolia* | 1 | 3 | 0 | 1 | 0 | 1 |
| PX776078.1 | *Veronica vandellioides* | 1 | 2 | 0 | 1 | 0 | 1 |
| PP840337.1 | *Veronica officinalis* | 1 | 2 | 0 | 1 | 0 | 1 |
| NC_056895.1 | *Veronicastrum axillare* | 1 | 2 | 0 | 0 | 1 | 1 |
| NC_082033.1 | *Veronicastrum brunonianum* | 0 | 3 | 0 | 1 | 1 | 1 |
| NC_069913.1 | *Veronicastrum latifolium* | 1 | 3 | 0 | 0 | 0 | 1 |
| NC_082029.1 | *Veronicastrum rhombifolium* | 1 | 3 | 0 | 1 | 1 | 1 |
| NC_082030.1 | *Veronicastrum robustum* | 1 | 3 | 0 | 1 | 1 | 1 |
| NC_082031.1 | *Veronicastrum stenostachyum* | 1 | 3 | 0 | 1 | 1 | 1 |
| NC_082032.1 | *Veronicastrum yunnanense* | 0 | 3 | 0 | 1 | 1 | 1 |
| NC_085506.1 | *[Verbascum songaricum](https://www.ncbi.nlm.nih.gov/nuccore/NC_085506.1)* | 0 | 1 | 0 | 1 | 0 | 3 |
| NC_085505.1 | *[Verbascum chaixii](https://www.ncbi.nlm.nih.gov/nuccore/NC_085505.1)* | 0 | 1 | 0 | 1 | 0 | 3 |
| NC_008535.1 | *Coffea arabica* | 5 | 0 | 0 | 0 | 0 | 3 |
| NC_007898_3 | *Solanum lycopersicum* | 5 | 0 | 0 | 1 | 0 | 0 |
| NC_008407.1 | *Jasminum nudiflorum* | 4 | 1 | 0 | 0 | 0 | 1 |
| MT108381.1 | *Avicennia marina* | 3 | 1 | 0 | 1 | 0 | 0 |
| MW174172.1 | *Acanthus ilicifolius* | 0 | 0 | 0 | 1 | 1 | 0 |
| NC_020431.1 | *Salvia miltiorrhiza* | 0 | 2 | 0 | 1 | 0 | 1 |
| NC_016433.1 | *Sesamum indicum* | 4 | 0 | 0 | 1 | 1 | 0 |
| MF579702 | *Forsythia suspensa* | 4 | 1 | 0 | 0 | 0 | 1 |

**Supplementary Table 3.** Nucleotide diversity (Pi) and hypervariable regions of plastid genomes

| Window Position | Midpoint Coordinate | Nucleotide Diversity (Pi) | Segregating Sites (S) | Corresponding Gene/Region |
| --- | --- | --- | --- | --- |
| 1-737 | 437 | 0.04833 | 42 | psbA |
| 321-937 | 637 | 0.02056 | 18 | psbA |
| 538-1137 | 837 | 0.01833 | 16 | psbA |
| 738-1337 | 1037 | 0.01444 | 13 | psbA |
| 938-1544 | 1237 | 0.01333 | 12 | psbA |
| 1138-1762 | 1437 | 0.02111 | 19 | psbA |
| 1338-1994 | 1658 | 0.03056 | 27 | psbA-trnK(exon2) |
| 1545-2195 | 1890 | 0.045 | 40 | trnK(exon2)-matK |
| 1763-2395 | 2095 | 0.03944 | 35 | matK |
| 1995-2595 | 2295 | 0.04944 | 44 | matK |
| 2196-2795 | 2495 | 0.05111 | 45 | matK |
| 2396-3001 | 2695 | 0.06278 | 55 | matK |
| 2596-3201 | 2895 | 0.06 | 53 | matK |
| 2796-3401 | 3101 | 0.05333 | 47 | matK |
| 3002-3621 | 3301 | 0.04167 | 37 | matK |
| 3202-3830 | 3501 | 0.035 | 31 | matK |
| 3402-4031 | 3721 | 0.02889 | 26 | matK-trnK(exon1) |
| 3622-4232 | 3931 | 0.03611 | 32 | matK-trnK(exon1) |
| 3831-4439 | 4132 | 0.04167 | 37 | matK-trnK(exon1) |
| 4032-4840 | 4333 | 0.07278 | 64 | trnK(exon1)-rps16(exon2) |
| 4233-5128 | 4547 | 0.105 | 93 | trnK(exon1)-rps16(exon2) |
| 4440-5328 | 5020 | 0.09889 | 87 | trnK(exon1)-rps16(exon2) |
| 4841-5562 | 5228 | 0.08 | 70 | rps16(exon2) |
| 5129-5769 | 5429 | 0.04611 | 40 | rps16(exon2)-rps16(exon1) |
| 5329-5972 | 5669 | 0.04444 | 39 | rps16(exon2)-rps16(exon1) |
| 5563-6179 | 5869 | 0.03222 | 29 | rps16(exon2)-rps16(exon1) |
| 5770-6386 | 6072 | 0.02556 | 23 | rps16(exon2)-rps16(exon1) |
| 5973-6651 | 6279 | 0.04056 | 36 | rps16(exon1)-trnQ |
| 6180-6916 | 6523 | 0.06056 | 54 | rps16(exon1)-trnQ |
| 6387-7124 | 6800 | 0.05944 | 53 | rps16(exon1)-trnQ |
| 6652-7331 | 7016 | 0.04278 | 38 | trnQ-psbK |
| 6917-7531 | 7224 | 0.01944 | 17 | trnQ-psbK |
| 7125-7744 | 7431 | 0.035 | 30 | psbK |
| 7332-7949 | 7634 | 0.03333 | 29 | psbK-psbI |
| 7532-8207 | 7847 | 0.04278 | 37 | psbK-psbI |
| 7745-8428 | 8049 | 0.035 | 31 | psbI-trnS |
| 7950-8640 | 8318 | 0.05944 | 52 | trnS-trnS-exon1 |
| 8208-8862 | 8538 | 0.07167 | 63 | trnS-trnS-exon1 |
| 8429-9076 | 8741 | 0.07889 | 68 | trnS-trnS-exon1 |
| 8641-9279 | 8962 | 0.06 | 52 | trnS-trnS-exon1 |
| 8863-9486 | 9176 | 0.05222 | 44 | trnS-exon1-trnS-exon2 |
| 9077-9695 | 9386 | 0.03944 | 34 | trnS-exon1-trnS-exon2 |
| 9280-9903 | 9587 | 0.05333 | 46 | trnS-exon1-trnS-exon2 |
| 9487-10112 | 9801 | 0.04833 | 43 | trnS-exon2-trnR |
| 9696-10317 | 10011 | 0.04778 | 42 | trnR |
| 9904-10517 | 10217 | 0.02611 | 23 | atpA |
| 10113-10717 | 10417 | 0.01833 | 16 | atpA |
| 10318-10917 | 10617 | 0.01556 | 14 | atpA |
| 10518-11117 | 10817 | 0.01556 | 14 | atpA |
| 10718-11317 | 11017 | 0.01444 | 13 | atpA |
| 10918-11517 | 11217 | 0.01222 | 11 | atpA |
| 11118-11732 | 11417 | 0.01889 | 17 | atpA |
| 11318-11932 | 11617 | 0.02778 | 25 | atpA |
| 11518-12132 | 11832 | 0.03222 | 29 | atpF(exon2) |
| 11733-12349 | 12032 | 0.03389 | 30 | atpF(exon2) |
| 11933-12554 | 12232 | 0.03056 | 27 | atpF(exon2)-atpF(exon1) |
| 12133-12758 | 12450 | 0.03389 | 30 | atpF(exon2)-atpF(exon1) |
| 12350-12965 | 12654 | 0.02111 | 19 | atpF(exon2)-atpF(exon1) |
| 12555-13240 | 12865 | 0.02333 | 21 | atpF(exon1) |
| 12759-13440 | 13140 | 0.01556 | 14 | atpF(exon1)-atpH |
| 12966-13640 | 13340 | 0.02111 | 19 | atpH |
| 13241-13864 | 13540 | 0.02444 | 22 | atpH |
| 13441-14305 | 13753 | 0.035 | 31 | atpH-atpI |
| 13641-14549 | 13978 | 0.03944 | 35 | atpH-atpI |
| 13865-14794 | 14434 | 0.03167 | 28 | atpH-atpI |
| 14306-14994 | 14694 | 0.02111 | 19 | atpI |
| 14550-15194 | 14894 | 0.01222 | 11 | atpI |
| 14795-15394 | 15094 | 0.01333 | 12 | atpI |
| 14995-15595 | 15294 | 0.03 | 27 | atpI |
| 15195-15795 | 15494 | 0.03444 | 31 | atpI-rps2 |
| 15395-15995 | 15695 | 0.03333 | 30 | rps2 |
| 15596-16195 | 15895 | 0.01667 | 15 | rps2 |
| 15796-16396 | 16095 | 0.01556 | 14 | rps2 |
| 15996-16628 | 16295 | 0.02778 | 24 | rps2 |
| 16196-16834 | 16511 | 0.03111 | 27 | rps2-rpoC2 |
| 16397-17034 | 16734 | 0.03333 | 29 | rpoC2 |
| 16629-17234 | 16934 | 0.02278 | 20 | rpoC2 |
| 16835-17434 | 17134 | 0.02167 | 19 | rpoC2 |
| 17035-17634 | 17334 | 0.02833 | 25 | rpoC2 |
| 17235-17834 | 17534 | 0.03444 | 31 | rpoC2 |
| 17435-18034 | 17734 | 0.04222 | 38 | rpoC2 |
| 17635-18234 | 17934 | 0.03556 | 32 | rpoC2 |
| 17835-18434 | 18134 | 0.03222 | 29 | rpoC2 |
| 18035-18634 | 18334 | 0.02389 | 21 | rpoC2 |
| 18235-18834 | 18534 | 0.02722 | 24 | rpoC2 |
| 18435-19034 | 18734 | 0.02278 | 20 | rpoC2 |
| 18635-19234 | 18934 | 0.03667 | 33 | rpoC2 |
| 18835-19434 | 19134 | 0.03 | 27 | rpoC2 |
| 19035-19634 | 19334 | 0.02889 | 26 | rpoC2 |
| 19235-19834 | 19534 | 0.01556 | 14 | rpoC2 |
| 19435-20034 | 19734 | 0.02556 | 23 | rpoC2 |
| 19635-20234 | 19934 | 0.02444 | 22 | rpoC2 |
| 19835-20434 | 20134 | 0.02111 | 19 | rpoC2 |
| 20035-20634 | 20334 | 0.00889 | 8 | rpoC2 |
| 20235-20834 | 20534 | 0.01444 | 13 | rpoC2 |
| 20435-21034 | 20734 | 0.01667 | 15 | rpoC2-rpoC1(exon2) |
| 20635-21234 | 20934 | 0.02333 | 21 | rpoC1(exon2) |
| 20835-21434 | 21134 | 0.01833 | 16 | rpoC1(exon2) |
| 21035-21634 | 21334 | 0.02056 | 18 | rpoC1(exon2) |
| 21235-21834 | 21534 | 0.02167 | 19 | rpoC1(exon2) |
| 21435-22034 | 21734 | 0.02222 | 20 | rpoC1(exon2) |
| 21635-22234 | 21934 | 0.02111 | 19 | rpoC1(exon2) |
| 21835-22434 | 22134 | 0.01722 | 15 | rpoC1(exon2) |
| 22035-22648 | 22334 | 0.02778 | 24 | rpoC1(exon2) |
| 22235-22873 | 22544 | 0.04222 | 37 | rpoC1(exon2)-rpoC1(exon1) |
| 22435-23078 | 22761 | 0.04889 | 43 | rpoC1(exon2)-rpoC1(exon1) |
| 22649-23283 | 22973 | 0.04056 | 36 | rpoC1(exon2)-rpoC1(exon1) |
| 22874-23483 | 23183 | 0.025 | 22 | rpoC1(exon2)-rpoC1(exon1) |
| 23079-23683 | 23383 | 0.02 | 18 | rpoC1(exon1) |
| 23284-23883 | 23583 | 0.01667 | 15 | rpoC1(exon1) |
| 23484-24083 | 23783 | 0.01444 | 13 | rpoB |
| 23684-24283 | 23983 | 0.01222 | 11 | rpoB |
| 23884-24483 | 24183 | 0.01556 | 14 | rpoB |
| 24084-24683 | 24383 | 0.01778 | 16 | rpoB |
| 24284-24883 | 24583 | 0.01444 | 13 | rpoB |
| 24484-25083 | 24783 | 0.01333 | 12 | rpoB |
| 24684-25283 | 24983 | 0.01667 | 15 | rpoB |
| 24884-25483 | 25183 | 0.02444 | 22 | rpoB |
| 25084-25683 | 25383 | 0.02333 | 21 | rpoB |
| 25284-25883 | 25583 | 0.02222 | 20 | rpoB |
| 25484-26083 | 25783 | 0.01667 | 15 | rpoB |
| 25684-26283 | 25983 | 0.01556 | 14 | rpoB |
| 25884-26483 | 26183 | 0.00889 | 8 | rpoB |
| 26084-26683 | 26383 | 0.01333 | 12 | rpoB |
| 26284-26883 | 26583 | 0.01278 | 11 | rpoB |
| 26484-27143 | 26783 | 0.05056 | 44 | rpoB |
| 26684-27405 | 26984 | 0.08833 | 77 | rpoB-trnC |
| 26884-27641 | 27295 | 0.10222 | 90 | rpoB-trnC |
| 27144-27851 | 27523 | 0.08056 | 71 | rpoB-trnC |
| 27406-28072 | 27749 | 0.05611 | 50 | rpoB-trnC |
| 27642-28285 | 27952 | 0.05333 | 47 | rpoB-trnC |
| 27852-28494 | 28185 | 0.06167 | 55 | rpoB-trnC |
| 28073-29137 | 28385 | 0.04611 | 41 | trnC-petN |
| 28286-29356 | 29037 | 0.04667 | 42 | trnC-petN |
| 28495-29617 | 29243 | 0.03667 | 33 | petN-psbM |
| 29138-29827 | 29495 | 0.04778 | 43 | petN-psbM |
| 29357-30032 | 29723 | 0.03444 | 31 | petN-psbM |
| 29618-30248 | 29932 | 0.03889 | 35 | psbM-trnD |
| 29828-30484 | 30132 | 0.03556 | 31 | psbM-trnD |
| 30033-30698 | 30376 | 0.04944 | 43 | psbM-trnD |
| 30249-30898 | 30591 | 0.035 | 30 | trnD-trnY |
| 30485-31118 | 30798 | 0.03833 | 34 | trnE |
| 30699-31344 | 31008 | 0.04833 | 43 | trnE-trnT |
| 30899-31554 | 31234 | 0.05167 | 46 | trnE-trnT |
| 31119-31768 | 31449 | 0.05667 | 50 | trnT |
| 31345-31977 | 31660 | 0.03833 | 34 | trnT-psbD |
| 31555-32185 | 31868 | 0.04944 | 43 | trnT-psbD |
| 31769-32418 | 32079 | 0.05667 | 50 | trnT-psbD |
| 31978-32618 | 32291 | 0.07778 | 69 | trnT-psbD |
| 32186-32827 | 32518 | 0.07 | 63 | trnT-psbD |
| 32419-33027 | 32727 | 0.04556 | 41 | trnT-psbD |
| 32619-33227 | 32927 | 0.02889 | 26 | psbD |
| 32828-33427 | 33127 | 0.02333 | 21 | psbD |
| 33028-33627 | 33327 | 0.02778 | 25 | psbD |
| 33228-33827 | 33527 | 0.01667 | 15 | psbD |
| 33428-34027 | 33727 | 0.01222 | 11 | psbD |
| 33628-34227 | 33927 | 0.01056 | 9 | psbC |
| 33828-34427 | 34127 | 0.01389 | 12 | psbC |
| 34028-34627 | 34327 | 0.01389 | 12 | psbC |
| 34228-34827 | 34527 | 0.01111 | 10 | psbC |
| 34428-35027 | 34727 | 0.01222 | 11 | psbC |
| 34628-35227 | 34927 | 0.01611 | 14 | psbC |
| 34828-35458 | 35127 | 0.02833 | 25 | psbC |
| 35028-35660 | 35327 | 0.03389 | 30 | psbC-trnS |
| 35228-35875 | 35560 | 0.04222 | 38 | trnS |
| 35459-36085 | 35761 | 0.03 | 27 | trnS-psbZ |
| 35661-36298 | 35985 | 0.03611 | 32 | trnS-psbZ |
| 35876-36511 | 36185 | 0.035 | 31 | psbZ-trnG |
| 36086-36730 | 36406 | 0.05444 | 48 | psbZ-trnG |
| 36299-36931 | 36620 | 0.04611 | 41 | trnG-trnM |
| 36512-37131 | 36830 | 0.03833 | 34 | trnM-rps14 |
| 36731-37331 | 37031 | 0.02444 | 22 | rps14 |
| 36932-37531 | 37231 | 0.02556 | 22 | rps14 |
| 37132-37731 | 37431 | 0.02111 | 18 | psaB |
| 37332-37931 | 37631 | 0.01556 | 13 | psaB |
| 37532-38131 | 37831 | 0.01111 | 10 | psaB |
| 37732-38331 | 38031 | 0.01333 | 12 | psaB |
| 37932-38531 | 38231 | 0.01556 | 14 | psaB |
| 38132-38731 | 38431 | 0.01222 | 11 | psaB |
| 38332-38931 | 38631 | 0.01333 | 12 | psaB |
| 38532-39131 | 38831 | 0.01444 | 13 | psaB |
| 38732-39331 | 39031 | 0.01778 | 16 | psaB |
| 38932-39531 | 39231 | 0.01667 | 15 | psaB |
| 39132-39731 | 39431 | 0.01 | 9 | psaB |
| 39332-39931 | 39631 | 0.00667 | 6 | psaA |
| 39532-40131 | 39831 | 0.00778 | 7 | psaA |
| 39732-40331 | 40031 | 0.01333 | 12 | psaA |
| 39932-40531 | 40231 | 0.01556 | 14 | psaA |
| 40132-40731 | 40431 | 0.01333 | 12 | psaA |
| 40332-40931 | 40631 | 0.01222 | 11 | psaA |
| 40532-41131 | 40831 | 0.01333 | 12 | psaA |
| 40732-41331 | 41031 | 0.01444 | 13 | psaA |
| 40932-41531 | 41231 | 0.01556 | 14 | psaA |
| 41132-41731 | 41431 | 0.02 | 18 | psaA |
| 41332-41931 | 41631 | 0.02556 | 23 | psaA |
| 41532-42132 | 41831 | 0.02778 | 25 | psaA |
| 41732-42375 | 42031 | 0.04556 | 41 | psaA-ycf3(exon3) |
| 41932-42648 | 42262 | 0.06333 | 57 | psaA-ycf3(exon3) |
| 42133-42851 | 42475 | 0.06444 | 58 | psaA-ycf3(exon3) |
| 42376-43057 | 42751 | 0.05444 | 49 | ycf3(exon3) |
| 42649-43257 | 42956 | 0.03222 | 29 | ycf3(exon3)-ycf3(exon2) |
| 42852-43458 | 43157 | 0.02889 | 26 | ycf3(exon3)-ycf3(exon2) |
| 43058-43671 | 43357 | 0.01889 | 17 | ycf3(exon3)-ycf3(exon2) |
| 43258-43871 | 43571 | 0.01556 | 14 | ycf3(exon2) |
| 43459-44080 | 43771 | 0.02222 | 20 | ycf3(exon2) |
| 43672-44280 | 43980 | 0.02333 | 21 | ycf3(exon2)-ycf3(exon1) |
| 43872-44490 | 44180 | 0.03556 | 32 | ycf3(exon2)-ycf3(exon1) |
| 44081-44707 | 44390 | 0.03111 | 28 | ycf3(exon2)-ycf3(exon1) |
| 44281-45479 | 44607 | 0.04722 | 42 | ycf3(exon1) |
| 44491-45681 | 45373 | 0.04778 | 42 | ycf3(exon1)-trnS |
| 44708-45918 | 45579 | 0.06722 | 59 | trnS |
| 45480-46118 | 45787 | 0.04889 | 43 | trnS-rps4 |
| 45682-46318 | 46018 | 0.03944 | 35 | rps4 |
| 45919-46518 | 46218 | 0.01889 | 17 | rps4 |
| 46119-46736 | 46418 | 0.02667 | 24 | rps4 |
| 46319-46960 | 46622 | 0.03444 | 31 | rps4-trnT |
| 46519-47316 | 46859 | 0.04778 | 43 | trnT |
| 46737-47550 | 47076 | 0.06167 | 54 | trnT-trnL-exon1 |
| 46961-47752 | 47439 | 0.05944 | 52 | trnT-trnL-exon1 |
| 47317-48026 | 47652 | 0.055 | 48 | trnL-exon1 |
| 47551-48227 | 47854 | 0.03667 | 33 | trnL-exon1-trnL-exon2 |
| 47753-48457 | 48127 | 0.05667 | 51 | trnL-exon1-trnL-exon2 |
| 48027-48663 | 48339 | 0.05389 | 48 | trnL-exon2-trnF |
| 48228-48898 | 48563 | 0.06278 | 56 | trnL-exon2-trnF |
| 48458-49153 | 48781 | 0.05944 | 52 | trnF-ndhJ |
| 48664-49353 | 49013 | 0.05556 | 49 | trnF-ndhJ |
| 48899-49553 | 49253 | 0.04444 | 39 | ndhJ |
| 49154-49756 | 49453 | 0.02556 | 23 | ndhJ |
| 49354-49956 | 49653 | 0.03 | 26 | ndhJ |
| 49554-50156 | 49856 | 0.03111 | 27 | ndhK |
| 49757-50356 | 50056 | 0.02722 | 23 | ndhK |
| 49957-50570 | 50256 | 0.01944 | 17 | ndhK |
| 50157-50770 | 50459 | 0.01778 | 15 | ndhK |
| 50357-50970 | 50670 | 0.01944 | 17 | ndhC |
| 50571-51179 | 50870 | 0.03667 | 32 | ndhC |
| 50771-51502 | 51070 | 0.07111 | 61 | ndhC-trnM |
| 50971-51707 | 51322 | 0.08889 | 76 | ndhC-trnM |
| 51180-51969 | 51602 | 0.09056 | 78 | ndhC-trnM |
| 51503-52193 | 51811 | 0.06 | 53 | ndhC-trnM |
| 51708-52394 | 52092 | 0.04556 | 41 | ndhC-trnM |
| 51970-52608 | 52293 | 0.02778 | 25 | ndhC-trnM |
| 52194-52875 | 52494 | 0.02778 | 25 | ndhC-trnM |
| 52395-53075 | 52708 | 0.02556 | 23 | ndhC-trnM |
| 52609-53294 | 52975 | 0.04222 | 38 | trnM |
| 52876-53494 | 53194 | 0.03667 | 33 | trnM-atpE |
| 53076-53694 | 53394 | 0.03889 | 35 | atpE |
| 53295-53894 | 53594 | 0.02 | 18 | atpE |
| 53495-54094 | 53794 | 0.01889 | 17 | atpB |
| 53695-54294 | 53994 | 0.01333 | 12 | atpB |
| 53895-54494 | 54194 | 0.01111 | 10 | atpB |
| 54095-54694 | 54394 | 0.01444 | 13 | atpB |
| 54295-54894 | 54594 | 0.01833 | 16 | atpB |
| 54495-55103 | 54794 | 0.02167 | 19 | atpB |
| 54695-55332 | 54994 | 0.03222 | 28 | atpB |
| 54895-55559 | 55211 | 0.04833 | 43 | atpB-rbcL |
| 55104-55762 | 55450 | 0.04833 | 43 | atpB-rbcL |
| 55333-55962 | 55659 | 0.03667 | 33 | atpB-rbcL |
| 55560-56162 | 55862 | 0.01944 | 17 | rbcL |
| 55763-56362 | 56062 | 0.01944 | 17 | rbcL |
| 55963-56562 | 56262 | 0.01833 | 16 | rbcL |
| 56163-56762 | 56462 | 0.01333 | 12 | rbcL |
| 56363-56962 | 56662 | 0.01111 | 10 | rbcL |
| 56563-57162 | 56862 | 0.01556 | 14 | rbcL |
| 56763-57398 | 57062 | 0.02944 | 26 | rbcL |
| 56963-57602 | 57283 | 0.03611 | 32 | rbcL-accD |
| 57163-57819 | 57498 | 0.045 | 39 | rbcL-accD |
| 57399-58027 | 57709 | 0.04333 | 38 | rbcL-accD |
| 57603-58227 | 57921 | 0.04556 | 40 | accD |
| 57820-58427 | 58127 | 0.03556 | 32 | accD |
| 58028-58636 | 58327 | 0.03111 | 28 | accD |
| 58228-58836 | 58536 | 0.02556 | 23 | accD |
| 58428-59036 | 58736 | 0.02444 | 22 | accD |
| 58637-59236 | 58936 | 0.02111 | 19 | accD |
| 58837-59445 | 59136 | 0.02556 | 23 | accD |
| 59037-59810 | 59336 | 0.03667 | 33 | accD |
| 59237-60032 | 59547 | 0.05389 | 48 | accD-psaI |
| 59446-60287 | 59916 | 0.05889 | 52 | accD-psaI |
| 59811-60514 | 60186 | 0.05 | 44 | accD-psaI |
| 60033-60730 | 60410 | 0.03833 | 34 | psaI-ycf4 |
| 60288-60930 | 60630 | 0.03 | 27 | psaI-ycf4 |
| 60515-61130 | 60830 | 0.03111 | 28 | ycf4 |
| 60731-61331 | 61030 | 0.03444 | 31 | ycf4 |
| 60931-61532 | 61230 | 0.05444 | 49 | ycf4 |
| 61131-61930 | 61431 | 0.07778 | 70 | ycf4-cemA |
| 61332-62141 | 61641 | 0.08333 | 75 | ycf4-cemA |
| 61533-62341 | 62040 | 0.06 | 54 | ycf4-cemA |
| 61931-62550 | 62241 | 0.03556 | 32 | cemA |
| 62142-62750 | 62450 | 0.02667 | 24 | cemA |
| 62342-62962 | 62650 | 0.02889 | 26 | cemA |
| 62551-63162 | 62850 | 0.02556 | 23 | cemA |
| 62751-63362 | 63062 | 0.02 | 18 | petA |
| 62963-63562 | 63262 | 0.02556 | 23 | petA |
| 63163-63762 | 63462 | 0.02889 | 26 | petA |
| 63363-63962 | 63662 | 0.02889 | 26 | petA |
| 63563-64174 | 63862 | 0.04389 | 39 | petA |
| 63763-64396 | 64062 | 0.05667 | 50 | petA-trnL(exon2) |
| 63963-64614 | 64296 | 0.08056 | 71 | petA-trnL(exon2) |
| 64175-64832 | 64509 | 0.06722 | 59 | trnL(exon2) |
| 64397-65039 | 64719 | 0.05389 | 47 | trnL(exon2)-psbJ |
| 64615-65244 | 64938 | 0.03444 | 30 | trnL(exon2)-psbJ |
| 64833-65444 | 65139 | 0.02167 | 19 | psbJ-trnL(exon1) |
| 65040-65644 | 65344 | 0.02611 | 23 | psbL-psbF |
| 65245-65845 | 65544 | 0.02222 | 19 | psbE |
| 65445-66345 | 65744 | 0.03222 | 28 | psbE-petL |
| 65645-66547 | 65946 | 0.03167 | 28 | psbE-petL |
| 65846-66764 | 66445 | 0.04278 | 38 | psbE-petL |
| 66346-66981 | 66648 | 0.04389 | 39 | petL |
| 66548-67193 | 66881 | 0.04722 | 42 | petL-petG |
| 66765-67427 | 67093 | 0.05444 | 49 | petG-trnW |
| 66982-67711 | 67327 | 0.05667 | 51 | trnW-trnP |
| 67194-67917 | 67527 | 0.05222 | 47 | trnP-psaJ |
| 67428-68153 | 67817 | 0.05111 | 45 | trnP-psaJ |
| 67712-68368 | 68036 | 0.05333 | 47 | psaJ-rpl33 |
| 67918-68580 | 68268 | 0.04667 | 41 | psaJ-rpl33 |
| 68160-68782 | 68480 | 0.03333 | 30 | rpl33 |
| 68369-68990 | 68680 | 0.02222 | 20 | rpl33-rps18 |
| 68581-69190 | 68890 | 0.02444 | 22 | rps18 |
| 68783-69392 | 69090 | 0.05333 | 48 | rps18 |
| 68991-69592 | 69292 | 0.05556 | 50 | rps18-rpl20 |
| 69191-69792 | 69492 | 0.05 | 45 | rpl20 |
| 69393-70005 | 69692 | 0.02556 | 23 | rpl20 |
| 69593-70205 | 69899 | 0.02889 | 26 | rpl20-rps12(exon1)/rps12-exon1 |
| 69793-70419 | 70105 | 0.03889 | 35 | rpl20-rps12(exon1)/rps12-exon1 |
| 70006-70627 | 70314 | 0.03667 | 33 | rpl20-rps12(exon1)/rps12-exon1 |
| 70206-70832 | 70526 | 0.04 | 36 | rpl20-rps12(exon1)/rps12-exon1 |
| 70420-71032 | 70727 | 0.03333 | 30 | rps12(exon1)/rps12-exon1-clpP(exon3) |
| 70628-71232 | 70932 | 0.03111 | 28 | clpP(exon3) |
| 70833-71444 | 71132 | 0.02222 | 20 | clpP(exon3)-clpP(exon2) |
| 71033-71666 | 71339 | 0.03056 | 27 | clpP(exon3)-clpP(exon2) |
| 71233-71867 | 71556 | 0.02389 | 21 | clpP(exon3)-clpP(exon2) |
| 71445-72067 | 71767 | 0.02722 | 24 | clpP(exon2) |
| 71667-72307 | 71967 | 0.03944 | 35 | clpP(exon2) |
| 71868-72513 | 72192 | 0.04722 | 42 | clpP(exon2)-clpP(exon1) |
| 72068-72714 | 72408 | 0.04389 | 39 | clpP(exon2)-clpP(exon1) |
| 72308-72945 | 72614 | 0.02222 | 20 | clpP(exon2)-clpP(exon1) |
| 72514-73157 | 72814 | 0.02111 | 19 | clpP(exon1) |
| 72715-73359 | 73057 | 0.02222 | 20 | clpP(exon1)-psbB |
| 72946-73559 | 73259 | 0.02667 | 24 | psbB |
| 73158-73759 | 73459 | 0.02778 | 25 | psbB |
| 73360-73959 | 73659 | 0.02889 | 26 | psbB |
| 73560-74159 | 73859 | 0.02778 | 25 | psbB |
| 73760-74359 | 74059 | 0.02444 | 22 | psbB |
| 73960-74559 | 74259 | 0.02333 | 21 | psbB |
| 74160-74759 | 74459 | 0.02 | 18 | psbB |
| 74360-74984 | 74659 | 0.03111 | 28 | psbB |
| 74560-75185 | 74871 | 0.03778 | 34 | psbB-psbT |
| 74760-75385 | 75084 | 0.03667 | 33 | psbT-psbN |
| 74985-75585 | 75285 | 0.02333 | 21 | psbN-psbH |
| 75186-75866 | 75485 | 0.01556 | 14 | psbH |
| 75386-76072 | 75766 | 0.03222 | 29 | psbH-petB-exon1 |
| 75586-76277 | 75966 | 0.04667 | 42 | petB-exon1-petB-exon2 |
| 75867-76487 | 76173 | 0.06111 | 55 | petB-exon1-petB-exon2 |
| 76073-76687 | 76378 | 0.04222 | 38 | petB-exon1-petB-exon2 |
| 76278-76887 | 76587 | 0.02722 | 24 | petB-exon2 |
| 76488-77087 | 76787 | 0.01167 | 10 | petB-exon2 |
| 76688-77293 | 76987 | 0.01722 | 15 | petB-exon2 |
| 76888-77498 | 77187 | 0.02333 | 21 | petB-exon2 |
| 77088-77698 | 77398 | 0.03222 | 29 | petD-exon1 |
| 77294-77908 | 77598 | 0.03667 | 33 | petD-exon1-petD-exon2 |
| 77499-78109 | 77808 | 0.03556 | 32 | petD-exon1-petD-exon2 |
| 77699-78309 | 78009 | 0.03 | 27 | petD-exon1-petD-exon2 |
| 77909-78509 | 78209 | 0.02667 | 24 | petD-exon2 |
| 78110-78773 | 78409 | 0.03333 | 30 | petD-exon2 |
| 78310-78977 | 78609 | 0.04111 | 37 | petD-exon2-rpoA |
| 78510-79177 | 78877 | 0.04111 | 37 | rpoA |
| 78774-79377 | 79077 | 0.04056 | 36 | rpoA |
| 78978-79577 | 79277 | 0.04056 | 36 | rpoA |
| 79178-79777 | 79477 | 0.03722 | 33 | rpoA |
| 79378-79982 | 79677 | 0.02778 | 25 | rpoA |
| 79578-80182 | 79882 | 0.02111 | 19 | rpoA-rps11 |
| 79778-80382 | 80082 | 0.02611 | 23 | rps11 |
| 79983-80582 | 80282 | 0.025 | 22 | rps11 |
| 80183-80782 | 80482 | 0.03278 | 29 | rpl36 |
| 80383-80982 | 80682 | 0.04167 | 37 | infA |
| 80583-81188 | 80882 | 0.04278 | 38 | infA-rps8 |
| 80783-81388 | 81088 | 0.03056 | 27 | rps8 |
| 80983-81598 | 81288 | 0.02778 | 25 | rps8 |
| 81189-81802 | 81497 | 0.03111 | 28 | rps8-rpl14 |
| 81389-82002 | 81702 | 0.03667 | 33 | rpl14 |
| 81599-82225 | 81902 | 0.03111 | 28 | rpl14 |
| 81803-82425 | 82125 | 0.02889 | 26 | rpl14-rpl16(exon2) |
| 82003-82640 | 82325 | 0.02556 | 23 | rpl16(exon2) |
| 82226-82840 | 82525 | 0.03222 | 29 | rpl16(exon2) |
| 82426-83056 | 82740 | 0.03444 | 31 | rpl16(exon2)-rpl16(exon1) |
| 82641-83264 | 82954 | 0.04 | 35 | rpl16(exon2)-rpl16(exon1) |
| 82841-83482 | 83160 | 0.05444 | 48 | rpl16(exon2)-rpl16(exon1) |
| 83057-83682 | 83375 | 0.055 | 48 | rpl16(exon1)-rps3 |
| 83265-83882 | 83582 | 0.04722 | 42 | rps3 |
| 83483-84082 | 83782 | 0.02833 | 25 | rps3 |
| 83683-84282 | 83982 | 0.03556 | 32 | rps3 |
| 83883-84482 | 84182 | 0.04167 | 37 | rpl22 |
| 84083-84682 | 84382 | 0.03833 | 34 | rpl22 |
| 84283-84882 | 84582 | 0.03222 | 28 | rpl22 |
| 84483-85106 | 84782 | 0.02722 | 24 | rps19 |
| 84683-85306 | 85006 | 0.01944 | 17 | rps19-rpl2(exon2) |
| 84883-85506 | 85206 | 0.00889 | 8 | rpl2(exon2) |
| 85107-85706 | 85406 | 0.00222 | 2 | rpl2(exon2) |
| 85307-85906 | 85606 | 0.005 | 4 | rpl2(exon2)-rpl2(exon1) |
| 85507-86106 | 85806 | 0.00611 | 5 | rpl2(exon2)-rpl2(exon1) |
| 85707-86306 | 86006 | 0.00722 | 6 | rpl2(exon2)-rpl2(exon1) |
| 85907-86506 | 86206 | 0.01 | 9 | rpl2(exon1) |
| 86107-86706 | 86406 | 0.00889 | 8 | rpl2(exon1) |
| 86307-86906 | 86606 | 0.00778 | 7 | rpl23 |
| 86507-87106 | 86806 | 0.00111 | 1 | rpl23-trnM |
| 86707-87306 | 87006 | 0.00333 | 3 | trnM |
| 86907-87506 | 87206 | 0.00778 | 7 | ycf2 |
| 87107-87706 | 87406 | 0.00778 | 7 | ycf2 |
| 87307-87906 | 87606 | 0.00556 | 5 | ycf2 |
| 87507-88112 | 87806 | 0.00389 | 3 | ycf2 |
| 87707-88312 | 88012 | 0.00833 | 7 | ycf2 |
| 87907-88623 | 88212 | 0.00944 | 8 | ycf2 |
| 88113-88823 | 88412 | 0.00889 | 8 | ycf2 |
| 88313-89023 | 88723 | 0.00667 | 6 | ycf2 |
| 88624-89232 | 88923 | 0.00778 | 7 | ycf2 |
| 88824-89432 | 89132 | 0.00889 | 8 | ycf2 |
| 89024-89632 | 89332 | 0.01222 | 11 | ycf2 |
| 89233-89832 | 89532 | 0.01222 | 11 | ycf2 |
| 89433-90032 | 89732 | 0.01222 | 11 | ycf2 |
| 89633-90232 | 89932 | 0.01222 | 11 | ycf2 |
| 89833-90432 | 90132 | 0.01222 | 11 | ycf2 |
| 90033-90632 | 90332 | 0.01111 | 10 | ycf2 |
| 90233-90832 | 90532 | 0.00889 | 8 | ycf2 |
| 90433-91032 | 90732 | 0.00833 | 7 | ycf2 |
| 90633-91232 | 90932 | 0.00611 | 5 | ycf2 |
| 90833-91435 | 91132 | 0.00722 | 6 | ycf2 |
| 91033-91635 | 91335 | 0.00556 | 5 | ycf2 |
| 91233-91835 | 91535 | 0.00667 | 6 | ycf2 |
| 91436-92035 | 91735 | 0.00333 | 3 | ycf2 |
| 91636-92235 | 91935 | 0.00333 | 3 | ycf2 |
| 91836-92435 | 92135 | 0.00333 | 3 | ycf2 |
| 92036-92635 | 92335 | 0.00333 | 3 | ycf2 |
| 92236-92835 | 92535 | 0.00556 | 5 | ycf2 |
| 92436-93041 | 92735 | 0.00444 | 4 | ycf2 |
| 92636-93241 | 92941 | 0.00889 | 8 | ycf2 |
| 92836-93441 | 93141 | 0.00667 | 6 | ycf2 |
| 93042-93641 | 93341 | 0.01 | 9 | ycf2 |
| 93242-93841 | 93541 | 0.00778 | 7 | ycf2 |
| 93442-94041 | 93741 | 0.00889 | 8 | ycf2 |
| 93642-94260 | 93941 | 0.01 | 9 | ycf2 |
| 93842-94469 | 94160 | 0.01556 | 14 | ycf2-trnL |
| 94042-94673 | 94360 | 0.01667 | 15 | ycf2-trnL |
| 94261-94873 | 94569 | 0.01444 | 13 | ycf2-trnL |
| 94470-95073 | 94773 | 0.00889 | 8 | trnL |
| 94674-95278 | 94973 | 0.00667 | 6 | trnL-ndhB(exon2) |
| 94874-95478 | 95178 | 0.00556 | 5 | trnL-ndhB(exon2) |
| 95074-95678 | 95378 | 0.00333 | 3 | ndhB(exon2) |
| 95279-95878 | 95578 | 0.00444 | 4 | ndhB(exon2) |
| 95479-96078 | 95778 | 0.00444 | 4 | ndhB(exon2) |
| 95679-96278 | 95978 | 0.01222 | 11 | ndhB(exon2) |
| 95879-96478 | 96178 | 0.01222 | 11 | ndhB(exon2)-ndhB(exon1) |
| 96079-96678 | 96378 | 0.01444 | 13 | ndhB(exon2)-ndhB(exon1) |
| 96279-96878 | 96578 | 0.00889 | 8 | ndhB(exon2)-ndhB(exon1) |
| 96479-97078 | 96778 | 0.00778 | 7 | ndhB(exon1) |
| 96679-97278 | 96978 | 0.00556 | 5 | ndhB(exon1) |
| 96879-97478 | 97178 | 0.00556 | 5 | ndhB(exon1) |
| 97079-97680 | 97378 | 0.01111 | 10 | ndhB(exon1) |
| 97279-97886 | 97580 | 0.01111 | 10 | ndhB(exon1)-rps7 |
| 97479-98086 | 97786 | 0.00889 | 8 | ndhB(exon1)-rps7 |
| 97681-98286 | 97986 | 0.00222 | 2 | rps7 |
| 97887-98486 | 98186 | 0.00111 | 1 | rps7 |
| 98087-98687 | 98386 | 0.00222 | 2 | rps12(exon3)-rps12(exon2) |
| 98287-98887 | 98587 | 0.00444 | 4 | rps12(exon3)-rps12(exon2) |
| 98487-99087 | 98787 | 0.00556 | 5 | rps12(exon3)-rps12(exon2) |
| 98688-99308 | 98987 | 0.00889 | 8 | rps12(exon2) |
| 98888-99518 | 99187 | 0.01444 | 13 | rps12(exon2)-trnV |
| 99088-99730 | 99417 | 0.01667 | 15 | rps12(exon2)-trnV |
| 99309-99943 | 99630 | 0.01667 | 15 | rps12(exon2)-trnV |
| 99519-100155 | 99837 | 0.01667 | 15 | rps12(exon2)-trnV |
| 99731-100360 | 100046 | 0.01778 | 16 | rps12(exon2)-trnV |
| 99944-100586 | 100255 | 0.01556 | 14 | rps12(exon2)-trnV |
| 100156-100786 | 100476 | 0.01222 | 11 | rps12(exon2)-trnV |
| 100361-100986 | 100686 | 0.00889 | 8 | trnV-rrn16S |
| 100587-101186 | 100886 | 0.00667 | 6 | rrn16S |
| 100787-101386 | 101086 | 0.00444 | 4 | rrn16S |
| 100987-101586 | 101286 | 0.00444 | 4 | rrn16S |
| 101187-101786 | 101486 | 0.00333 | 3 | rrn16S |
| 101387-101986 | 101686 | 0.00444 | 4 | rrn16S |
| 101587-102186 | 101886 | 0.00333 | 3 | rrn16S |
| 101787-102386 | 102086 | 0.00333 | 3 | rrn16S |
| 101987-102586 | 102286 | 0.00556 | 5 | rrn16S |
| 102187-102786 | 102486 | 0.00556 | 5 | rrn16S-trnI-exon1 |
| 102387-102986 | 102686 | 0.00778 | 7 | trnI-exon1-trnI-exon2 |
| 102587-103187 | 102886 | 0.00889 | 8 | trnI-exon1-trnI-exon2 |
| 102787-103387 | 103086 | 0.01222 | 11 | trnI-exon1-trnI-exon2 |
| 102987-103587 | 103287 | 0.01333 | 12 | trnI-exon1-trnI-exon2 |
| 103188-103787 | 103487 | 0.00667 | 6 | trnI-exon1-trnI-exon2 |
| 103388-103987 | 103687 | 0.00333 | 3 | trnI-exon2-trnA-exon1 |
| 103588-104187 | 103887 | 0.00111 | 1 | trnA-exon1-trnA-exon2 |
| 103788-104396 | 104087 | 0.00444 | 4 | trnA-exon1-trnA-exon2 |
| 103988-104596 | 104287 | 0.00556 | 5 | trnA-exon1-trnA-exon2 |
| 104188-104796 | 104496 | 0.00444 | 4 | trnA-exon1-trnA-exon2 |
| 104397-104996 | 104696 | 0.00222 | 2 | trnA-exon2-rrn23S |
| 104597-105196 | 104896 | 0.00111 | 1 | rrn23S |
| 104797-105396 | 105096 | 0.00111 | 1 | rrn23S |
| 104997-105596 | 105296 | 0 | 0 | rrn23S |
| 105197-105796 | 105496 | 0.00222 | 2 | rrn23S |
| 105397-105996 | 105696 | 0.00222 | 2 | rrn23S |
| 105597-106196 | 105896 | 0.00333 | 3 | rrn23S |
| 105797-106396 | 106096 | 0.00111 | 1 | rrn23S |
| 105997-106596 | 106296 | 0.00111 | 1 | rrn23S |
| 106197-106796 | 106496 | 0 | 0 | rrn23S |
| 106397-106996 | 106696 | 0.00111 | 1 | rrn23S |
| 106597-107196 | 106896 | 0.00111 | 1 | rrn23S |
| 106797-107396 | 107096 | 0.00111 | 1 | rrn23S |
| 106997-107596 | 107296 | 0 | 0 | rrn23S |
| 107197-107796 | 107496 | 0.00111 | 1 | rrn23S |
| 107397-107996 | 107696 | 0.00222 | 2 | rrn4.5S |
| 107597-108203 | 107896 | 0.00444 | 4 | rrn4.5S-rrn5S |
| 107797-108403 | 108096 | 0.01333 | 12 | rrn5S |
| 107997-108617 | 108303 | 0.02056 | 18 | rrn5S-trnR |
| 108204-108826 | 108504 | 0.03278 | 29 | trnR-trnN |
| 108404-109032 | 108726 | 0.02944 | 26 | trnR-trnN |
| 108618-109232 | 108926 | 0.02556 | 23 | trnR-trnN |
| 108827-109433 | 109132 | 0.01333 | 12 | trnN-ndhF |
| 109033-109633 | 109333 | 0.00778 | 7 | trnN-ndhF |
| 109233-109833 | 109533 | 0.00778 | 7 | trnN-ndhF |
| 109434-110033 | 109733 | 0.00778 | 7 | trnN-ndhF |
| 109634-110233 | 109933 | 0.01278 | 11 | trnN-ndhF |
| 109834-110433 | 110133 | 0.01611 | 14 | trnN-ndhF |
| 110034-110633 | 110333 | 0.025 | 22 | trnN-ndhF |
| 110234-110836 | 110533 | 0.02889 | 26 | trnN-ndhF |
| 110434-111036 | 110736 | 0.03722 | 33 | ndhF |
| 110634-111236 | 110936 | 0.045 | 40 | ndhF |
| 110837-111442 | 111136 | 0.05944 | 53 | ndhF |
| 111037-111642 | 111336 | 0.05667 | 51 | ndhF |
| 111237-111842 | 111542 | 0.05167 | 46 | ndhF |
| 111443-112042 | 111742 | 0.03278 | 29 | ndhF |
| 111643-112242 | 111942 | 0.03222 | 28 | ndhF |
| 111843-112442 | 112142 | 0.025 | 22 | ndhF |
| 112043-112642 | 112342 | 0.02611 | 23 | ndhF |
| 112243-112842 | 112542 | 0.02444 | 22 | ndhF |
| 112443-113047 | 112742 | 0.03444 | 31 | ndhF |
| 112643-113270 | 112942 | 0.055 | 49 | ndhF-rpl32 |
| 112843-113633 | 113147 | 0.07444 | 66 | ndhF-rpl32 |
| 113048-113835 | 113518 | 0.07611 | 67 | ndhF-rpl32 |
| 113271-114066 | 113733 | 0.08611 | 75 | rpl32 |
| 113634-114429 | 113945 | 0.10278 | 89 | rpl32-trnL |
| 113836-114642 | 114194 | 0.11778 | 102 | rpl32-trnL |
| 114067-114853 | 114541 | 0.08833 | 77 | rpl32-trnL |
| 114430-115059 | 114748 | 0.05778 | 51 | trnL |
| 114643-115259 | 114959 | 0.04111 | 37 | ccsA |
| 114854-115459 | 115159 | 0.05111 | 46 | ccsA |
| 115060-115659 | 115359 | 0.05111 | 45 | ccsA |
| 115260-115865 | 115559 | 0.04944 | 43 | ccsA |
| 115460-116087 | 115759 | 0.05222 | 45 | ccsA |
| 115660-116287 | 115970 | 0.04556 | 40 | ccsA-ndhD |
| 115866-116487 | 116187 | 0.03833 | 33 | ndhD |
| 116088-116687 | 116387 | 0.02889 | 25 | ndhD |
| 116288-116887 | 116587 | 0.02889 | 25 | ndhD |
| 116488-117087 | 116787 | 0.02778 | 25 | ndhD |
| 116688-117287 | 116987 | 0.02222 | 20 | ndhD |
| 116888-117487 | 117187 | 0.02333 | 21 | ndhD |
| 117088-117695 | 117387 | 0.03389 | 30 | ndhD |
| 117288-117895 | 117587 | 0.03722 | 33 | ndhD |
| 117488-118111 | 117795 | 0.04722 | 42 | psaC |
| 117696-118317 | 117995 | 0.04667 | 42 | psaC-ndhE |
| 117896-118517 | 118217 | 0.04333 | 39 | psaC-ndhE |
| 118112-118733 | 118417 | 0.05 | 45 | ndhE |
| 118318-118938 | 118617 | 0.03778 | 34 | ndhE-ndhG |
| 118518-119138 | 118838 | 0.03889 | 35 | ndhG |
| 118734-119340 | 119038 | 0.02556 | 22 | ndhG |
| 118939-119563 | 119238 | 0.04778 | 42 | ndhG |
| 119139-119794 | 119453 | 0.06556 | 57 | ndhG-ndhI |
| 119341-119994 | 119694 | 0.05889 | 52 | ndhG-ndhI |
| 119564-120194 | 119894 | 0.03667 | 32 | ndhI |
| 119795-120405 | 120094 | 0.02222 | 20 | ndhI |
| 119995-120605 | 120304 | 0.02667 | 24 | ndhI-ndhA(exon2) |
| 120195-120805 | 120505 | 0.03 | 27 | ndhA(exon2) |
| 120406-121011 | 120705 | 0.02667 | 24 | ndhA(exon2) |
| 120606-121241 | 120905 | 0.03 | 27 | ndhA(exon2)-ndhA(exon1) |
| 120806-121445 | 121116 | 0.05778 | 52 | ndhA(exon2)-ndhA(exon1) |
| 121012-121648 | 121345 | 0.06444 | 58 | ndhA(exon2)-ndhA(exon1) |
| 121242-121872 | 121545 | 0.06778 | 61 | ndhA(exon2)-ndhA(exon1) |
| 121446-122080 | 121764 | 0.04 | 36 | ndhA(exon2)-ndhA(exon1) |
| 121649-122280 | 121980 | 0.03222 | 29 | ndhA(exon1) |
| 121873-122480 | 122180 | 0.02167 | 19 | ndhA(exon1) |
| 122081-122680 | 122380 | 0.02278 | 19 | ndhA(exon1) |
| 122281-122880 | 122580 | 0.03167 | 27 | ndhH |
| 122481-123080 | 122780 | 0.02944 | 25 | ndhH |
| 122681-123280 | 122980 | 0.02833 | 25 | ndhH |
| 122881-123480 | 123180 | 0.02167 | 19 | ndhH |
| 123081-123680 | 123380 | 0.02333 | 21 | ndhH |
| 123281-123881 | 123580 | 0.02667 | 24 | ndhH |
| 123481-124084 | 123781 | 0.03333 | 30 | ndhH-rps15 |
| 123681-124299 | 123981 | 0.06278 | 55 | rps15 |
| 123882-124619 | 124198 | 0.09667 | 84 | rps15-ycf1 |
| 124085-124819 | 124426 | 0.10111 | 88 | rps15-ycf1 |
| 124300-125019 | 124719 | 0.08167 | 72 | ycf1 |
| 124620-125219 | 124919 | 0.05611 | 50 | ycf1 |
| 124820-125419 | 125119 | 0.05722 | 51 | ycf1 |
| 125020-125619 | 125319 | 0.05278 | 47 | ycf1 |
| 125220-125840 | 125519 | 0.06389 | 57 | ycf1 |
| 125420-126055 | 125740 | 0.07167 | 64 | ycf1 |
| 125620-126261 | 125946 | 0.08444 | 75 | ycf1 |
| 125841-126461 | 126161 | 0.08 | 71 | ycf1 |
| 126056-126667 | 126361 | 0.07389 | 65 | ycf1 |
| 126262-126867 | 126567 | 0.07056 | 62 | ycf1 |
| 126462-127163 | 126767 | 0.07556 | 67 | ycf1 |
| 126668-127363 | 126967 | 0.07556 | 67 | ycf1 |
| 126868-127563 | 127263 | 0.06722 | 60 | ycf1 |
| 127164-127763 | 127463 | 0.05556 | 49 | ycf1 |
| 127364-127963 | 127663 | 0.05278 | 47 | ycf1 |
| 127564-128172 | 127863 | 0.05833 | 51 | ycf1 |
| 127764-128378 | 128072 | 0.07389 | 65 | ycf1 |
| 127964-128584 | 128278 | 0.08722 | 76 | ycf1 |
| 128173-128787 | 128478 | 0.08611 | 76 | ycf1 |
| 128379-128987 | 128684 | 0.06333 | 56 | ycf1 |
| 128585-129187 | 128887 | 0.03889 | 35 | ycf1 |
| 128788-129387 | 129087 | 0.025 | 22 | ycf1 |
| 128988-129587 | 129287 | 0.015 | 13 | ycf1-trnN |
| 129188-129787 | 129487 | 0.01056 | 9 | ycf1-trnN |
| 129388-129987 | 129687 | 0.00667 | 6 | ycf1-trnN |
| 129588-130188 | 129887 | 0.00667 | 6 | ycf1-trnN |
| 129788-130394 | 130088 | 0.00889 | 8 | ycf1-trnN |
| 129988-130594 | 130288 | 0.01889 | 17 | trnN |
| 130189-130816 | 130494 | 0.03056 | 27 | trnN-trnR |
| 130395-131017 | 130694 | 0.02944 | 26 | trnN-trnR |
| 130595-131224 | 130917 | 0.025 | 22 | trnR |
| 130817-131424 | 131117 | 0.01222 | 11 | trnR-rrn5S |
| 131018-131624 | 131324 | 0.01 | 9 | rrn5S |
| 131225-131824 | 131524 | 0.00333 | 3 | rrn5S-rrn4.5S |
| 131425-132024 | 131724 | 0.00222 | 2 | rrn4.5S-rrn23S |
| 131625-132224 | 131924 | 0 | 0 | rrn23S |
| 131825-132424 | 132124 | 0.00111 | 1 | rrn23S |
| 132025-132624 | 132324 | 0.00111 | 1 | rrn23S |
| 132225-132824 | 132524 | 0.00111 | 1 | rrn23S |
| 132425-133024 | 132724 | 0 | 0 | rrn23S |
| 132625-133224 | 132924 | 0 | 0 | rrn23S |
| 132825-133424 | 133124 | 0.00111 | 1 | rrn23S |
| 133025-133624 | 133324 | 0.00111 | 1 | rrn23S |
| 133225-133824 | 133524 | 0.00333 | 3 | rrn23S |
| 133425-134024 | 133724 | 0.00222 | 2 | rrn23S |
| 133625-134224 | 133924 | 0.00222 | 2 | rrn23S |
| 133825-134424 | 134124 | 0.00111 | 1 | rrn23S |
| 134025-134624 | 134324 | 0.00111 | 1 | rrn23S |
| 134225-134824 | 134524 | 0.00111 | 1 | rrn23S |
| 134425-135033 | 134724 | 0.00111 | 1 | rrn23S-trnA(exon2) |
| 134625-135233 | 134924 | 0.00444 | 4 | trnA(exon2)-trnA(exon1) |
| 134825-135433 | 135133 | 0.00556 | 5 | trnA(exon2)-trnA(exon1) |
| 135034-135633 | 135333 | 0.00444 | 4 | trnA(exon2)-trnA(exon1) |
| 135234-135833 | 135533 | 0.00222 | 2 | trnA(exon2)-trnA(exon1) |
| 135434-136033 | 135733 | 0.00444 | 4 | trnI(exon2) |
| 135634-136233 | 135933 | 0.00667 | 6 | trnI(exon2)-trnI(exon1) |
| 135834-136434 | 136133 | 0.01222 | 11 | trnI(exon2)-trnI(exon1) |
| 136034-136634 | 136334 | 0.01111 | 10 | trnI(exon2)-trnI(exon1) |
| 136234-136834 | 136534 | 0.00889 | 8 | trnI(exon2)-trnI(exon1) |
| 136435-137034 | 136734 | 0.00778 | 7 | trnI(exon1) |
| 136635-137234 | 136934 | 0.00556 | 5 | trnI(exon1)-rrn16S |
| 136835-137434 | 137134 | 0.00556 | 5 | rrn16S |
| 137035-137634 | 137334 | 0.00333 | 3 | rrn16S |
| 137235-137834 | 137534 | 0.00333 | 3 | rrn16S |
| 137435-138034 | 137734 | 0.00444 | 4 | rrn16S |
| 137635-138234 | 137934 | 0.00333 | 3 | rrn16S |
| 137835-138434 | 138134 | 0.00444 | 4 | rrn16S |
| 138035-138634 | 138334 | 0.00556 | 5 | rrn16S |
| 138235-138834 | 138534 | 0.00667 | 6 | rrn16S |
| 138435-139060 | 138734 | 0.01 | 9 | rrn16S-trnV |
| 138635-139274 | 138953 | 0.01444 | 13 | trnV-rps12-exon2 |
| 138835-139477 | 139165 | 0.01667 | 15 | trnV-rps12-exon2 |
| 139061-139690 | 139377 | 0.01667 | 15 | trnV-rps12-exon2 |
| 139275-139903 | 139590 | 0.01444 | 13 | trnV-rps12-exon2 |
| 139478-140112 | 139799 | 0.01889 | 17 | trnV-rps12-exon2 |
| 139691-140333 | 140012 | 0.01667 | 15 | trnV-rps12-exon2 |
| 139904-140533 | 140233 | 0.01333 | 12 | trnV-rps12-exon2 |
| 140113-140733 | 140433 | 0.00778 | 7 | rps12-exon2 |
| 140334-140934 | 140633 | 0.00556 | 5 | rps12-exon2-rps12-exon3 |
| 140534-141134 | 140833 | 0.00444 | 4 | rps12-exon2-rps12-exon3 |
| 140734-141334 | 141034 | 0 | 0 | rps12-exon3 |
| 140935-141534 | 141234 | 0.00111 | 1 | rps7 |
| 141135-141740 | 141434 | 0.00333 | 3 | rps7 |
| 141335-141942 | 141640 | 0.01111 | 10 | rps7-ndhB-exon1 |
| 141535-142142 | 141842 | 0.01111 | 10 | rps7-ndhB-exon1 |
| 141741-142342 | 142042 | 0.01111 | 10 | ndhB-exon1 |
| 141943-142542 | 142242 | 0.00333 | 3 | ndhB-exon1 |
| 142143-142742 | 142442 | 0.00889 | 8 | ndhB-exon1 |
| 142343-142942 | 142642 | 0.00667 | 6 | ndhB-exon1-ndhB-exon2 |
| 142543-143142 | 142842 | 0.01111 | 10 | ndhB-exon1-ndhB-exon2 |
| 142743-143342 | 143042 | 0.01111 | 10 | ndhB-exon1-ndhB-exon2 |
| 142943-143542 | 143242 | 0.01222 | 11 | ndhB-exon1-ndhB-exon2 |
| 143143-143742 | 143442 | 0.01 | 9 | ndhB-exon2 |
| 143343-143942 | 143642 | 0.00444 | 4 | ndhB-exon2 |
| 143543-144142 | 143842 | 0.00444 | 4 | ndhB-exon2 |
| 143743-144347 | 144042 | 0.00333 | 3 | ndhB-exon2 |
| 143943-144547 | 144247 | 0.00667 | 6 | ndhB-exon2-trnL |
| 144143-144751 | 144447 | 0.00667 | 6 | ndhB-exon2-trnL |
| 144348-144960 | 144647 | 0.01222 | 11 | trnL |
| 144548-145160 | 144851 | 0.01556 | 14 | trnL-ycf2 |
| 144752-145379 | 145060 | 0.01667 | 15 | trnL-ycf2 |
| 144961-145579 | 145279 | 0.01222 | 11 | trnL-ycf2 |
| 145161-145779 | 145479 | 0.00778 | 7 | ycf2 |
| 145380-145979 | 145679 | 0.01 | 9 | ycf2 |
| 145580-146179 | 145879 | 0.01 | 9 | ycf2 |
| 145780-146379 | 146079 | 0.01 | 9 | ycf2 |
| 145980-146585 | 146279 | 0.00556 | 5 | ycf2 |
| 146180-146785 | 146479 | 0.00667 | 6 | ycf2 |
| 146380-146985 | 146685 | 0.00556 | 5 | ycf2 |
| 146586-147185 | 146885 | 0.00556 | 5 | ycf2 |
| 146786-147385 | 147085 | 0.00333 | 3 | ycf2 |
| 146986-147585 | 147285 | 0.00222 | 2 | ycf2 |
| 147186-147785 | 147485 | 0.00444 | 4 | ycf2 |
| 147386-147985 | 147685 | 0.00667 | 6 | ycf2 |
| 147586-148188 | 147885 | 0.00667 | 6 | ycf2 |
| 147786-148388 | 148088 | 0.00444 | 4 | ycf2 |
| 147986-148588 | 148288 | 0.005 | 4 | ycf2 |
| 148189-148788 | 148488 | 0.00611 | 5 | ycf2 |
| 148389-148988 | 148688 | 0.00833 | 7 | ycf2 |
| 148589-149188 | 148888 | 0.00778 | 7 | ycf2 |
| 148789-149388 | 149088 | 0.01111 | 10 | ycf2 |
| 148989-149588 | 149288 | 0.01222 | 11 | ycf2 |
| 149189-149788 | 149488 | 0.01444 | 13 | ycf2 |
| 149389-149988 | 149688 | 0.01222 | 11 | ycf2 |
| 149589-150188 | 149888 | 0.01556 | 14 | ycf2 |
| 149789-150397 | 150088 | 0.01 | 9 | ycf2 |
| 149989-150597 | 150297 | 0.00889 | 8 | ycf2 |
| 150189-150797 | 150497 | 0.00556 | 5 | ycf2 |
| 150398-151108 | 150697 | 0.00778 | 7 | ycf2 |
| 150598-151308 | 151008 | 0.01 | 9 | ycf2 |
| 150798-151514 | 151208 | 0.00833 | 7 | ycf2 |
| 151109-151714 | 151414 | 0.00722 | 6 | ycf2 |
| 151309-151914 | 151614 | 0.00389 | 3 | ycf2 |
| 151515-152114 | 151814 | 0.00667 | 6 | ycf2 |
| 151715-152314 | 152014 | 0.00778 | 7 | ycf2 |
| 151915-152514 | 152214 | 0.00667 | 6 | ycf2 |
| 152115-152714 | 152414 | 0.00222 | 2 | trnM-rpl23 |
| 152315-152914 | 152614 | 0.00111 | 1 | rpl23 |
| 152515-153114 | 152814 | 0.00778 | 7 | rpl23 |
| 152715-153314 | 153014 | 0.01 | 9 | rpl2-exon1 |
| 152915-153514 | 153214 | 0.01 | 9 | rpl2-exon1 |
| 153115-153714 | 153414 | 0.00722 | 6 | rpl2-exon1-rpl2-exon2 |
| 153315-153914 | 153614 | 0.005 | 4 | rpl2-exon1-rpl2-exon2 |
| 153515-154114 | 153814 | 0.005 | 4 | rpl2-exon1-rpl2-exon2 |
| 153715-154314 | 154014 | 0.00222 | 2 | rpl2-exon2 |
| 153915-154402 | 154153 | 0.00558 | 4 | rpl2-exon2 |
